# Connecting Brown Carbon Composition and Physicochemical Properties of Aqueous Urban PM_2.5_ to their Photosensitized Production of Singlet Oxygen and Organic Triplet Excited States

**DOI:** 10.1021/acs.est.5c15686

**Published:** 2026-02-14

**Authors:** Yuting Lyu, Yitao Li, Ruihan Ma, Tianye Zhou, Nadine Borduas-Dedekind, Theodora Nah

**Affiliations:** † School of Energy and Environment, City University of Hong Kong, Kowloon, Hong Kong SAR 999077, China; ‡ State Key Laboratory of Marine Environmental Health, City University of Hong Kong, Kowloon, Hong Kong SAR 999077, China; § Department of Chemistry, 8166 University of British Columbia, Vancouver, British Columbia V6T 1Z1, Canada

**Keywords:** urban PM_2.5_, photochemistry, brown carbon, organic triplet excited states, singlet oxygen

## Abstract

Oxidants, including singlet oxygen (^1^O_2_*) and organic triplet excited states (^3^C*) formed from the photoexcitation of brown carbon (BrC), drive many chemical processes in atmospheric waters. However, due to the chemical complexity of atmospheric BrC, many questions remain about the specific BrC chromophores and physicochemical properties that primarily control ^1^O_2_* and ^3^C* production. In this study, we present a framework for apportioning photosensitizers and predicting the production of ^1^O_2_* and ^3^C* based on measurable physicochemical properties of BrC. This is achieved by combining photochemical experiments with absorbance and fluorescence measurements and statistical modeling of a year-long data set of PM_2.5_ extracts from Hong Kong SAR, China. Parallel Factor and Non-negative Matrix Factorization analyses of the fluorescence data revealed that highly oxygenated organic aerosols were the main contributors to ^1^O_2_* production, whereas less oxygenated organic aerosols were the main contributors to ^3^C* production. Next, we developed Orthogonal Partial Least Squares-Multiple Linear Regression models that successfully predicted ^1^O_2_* and ^3^C* steady-state concentrations ([^1^O_2_*]_ss_ and [^3^C*]_ss_) and quantum yields ( ΦO2*1 and Φ_^3^C*_) from standard optical measurements. These models revealed that while [^1^O_2_*]_ss_ and [^3^C*]_ss_ depended on parameters that reflected the quantities of BrC chromophores, ΦO2*1 and Φ_^3^C*_ were influenced by the specific types (i.e., quality) of BrC chromophores present. Overall, this combined approach provides a powerful tool for identifying key BrC chromophore components and specific physicochemical properties that drive ^1^O_2_* and ^3^C* production.

## Graphical abstract

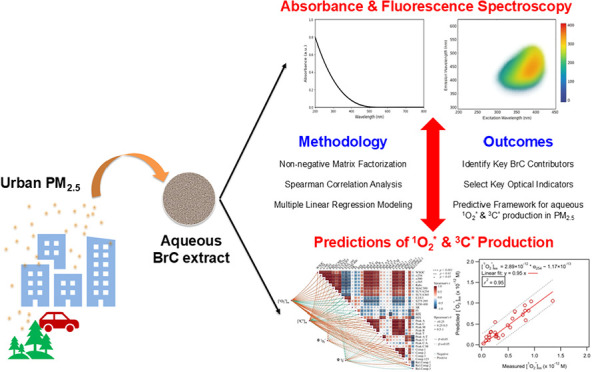

## 1 Introduction

Atmospheric brown carbon (BrC), the chromophoric fraction of organic aerosols, play significant roles in climate and atmospheric chemistry.^1–3^ Some BrC chromophores can act as photosensitizers that produce a variety of oxidants upon photon absorption.^3–5^ During photosensitization, the primary reactive species formed are organic triplet excited states (^3^C*), which are generated after light absorption and intersystem crossing. ^3^C* can then produce secondary oxidants, such as singlet oxygen (^1^O_2_*) via energy transfer to molecular oxygen, as well as radicals such as hydroxyl (^•^OH) and superoxide (O_2_^•–^) radicals through electron-transfer reactions. These oxidants react with aerosol components to alter the aerosol composition and physicochemical properties, and consequently their reactivities and atmospheric lifetimes. ^1^O_2_* and the chemically complex pool of ^3^C* have been receiving increasing attention due to their high concentrations in atmospheric aqueous phases (e.g., cloud and fog droplets, aqueous aerosols) and high aqueous-phase reactivities with olefinic compounds (e.g., alkenoates, alkenols, dienes), amino acids, phenolic compounds, carboxamides, carboxylic acids, and carboxamides.^6–8^ Steady-state concentrations of ^1^O_2_* ([^1^O_2_*]_ss_) and ^3^C* ([^3^C*]_ss_) in atmospheric aqueous phases typically range from 10^–15^ to 10^–12^ M and from 10^–16^ to 10^–13^ M, respectively.^8–19^ While ^•^OH are seen as major aqueous-phase oxidants due to their nonselective and highly reactive nature, ^1^O_2_* and ^3^C* concentrations in atmospheric aqueous phases can be high enough to offset their lower reactivities, leading to ^1^O_2_* and ^3^C* being the main aqueous-phase oxidants for many classes of organic compounds.^7,8^ Previously reported quantum yields of ^1^O_2_* ( ΦO2*1 ) and ^3^C* ( ΦC*3 ) for atmospheric aqueous BrC, which describe the efficiencies of ^1^O_2_* and ^3^C* photosensitization, range from 0.3 to 13.4% and from 0.05 to 4.1%, respectively.^7–18^

The conventional method for determining [^1^O_2_*]_ss_, [^3^C*]_ss_, ΦO2*1 , and ΦC*3 involves probe-based photochemical experiments.^20,21^ The aqueous BrC sample or extract is first mixed with a specific probe and irradiated with simulated sunlight. [^1^O_2_*]_ss_, [^3^C*]_ss_, ΦO2*1 , and ΦC*3 are derived from the decay rates of the probes and absorption rates of the mixture. This experimental process can be time-consuming and labor-intensive, especially for long-term field studies that involve a large number of ambient samples and aim to investigate [^1^O_2_*]_ss_, [^3^C*]_ss_, ΦO2*1 , and ΦC*3 at high temporal resolution. Hence, there is a need for methods that can determine [^1^O_2_*]_ss_, [^3^C*]_ss_, ΦO2*1 , and ΦC*3 for aqueous BrC without requiring extensive photochemistry experiments.

One possible method for determining [^1^O_2_*]_ss_, [^3^C*]_ss_, ΦO2*1 , and ΦC*3 involves linking the physicochemical properties of BrC with their ^1^O_2_* and ^3^C* production. Absorbance and fluorescence parameters can serve as proxies for specific physicochemical properties of chromophoric organic matter.^22^ Previous studies have reported correlations between some of these absorbance and fluorescence parameters and [^1^O_2_*]_ss_, [^3^C*]_ss_, ΦO2*1 , and ΦC*3 for aqueous BrC. For example, the [^1^O_2_*]_ss_ and [^3^C*]_ss_ for aqueous BrC extracts from PM_10_ filters in Grenoble (France) exhibited negative correlations with the *E*_2_/*E*_3_ ratio (i.e., ratio of absorbance at 254 and 365 nm).^7^ ΦO2*1 was also positively correlated with *E*_2_/*E*_3_ whereas ΦC*3 had weak to no correlations with *E*_2_/*E*_3_,^7^ similar to observations made for aqueous BrC extracts from PM_2.5_ in Davis (California).^23^ Since an increase in *E*_2_/*E*_3_ is typically indicative of a decrease in molecular weight, these results indicated that both [^1^O_2_*]_ss_ and [^3^C*]_ss_ decreased when the molecular weight of BrC species decreased, whereas the converse occurred for ΦO2*1 . [^1^O_2_*]_ss_ was positively correlated to the mass absorption coefficient at 300 nm (MAC_300_) and specific UV absorbance at 254 nm (SUVA_254_) for aqueous BrC extracts from PM filters in Roveredo (Switzerland) and Hong Kong (China SAR).^8,16^ MAC_300_ and SUVA_254_, whereby higher values typically indicate higher aromaticity, were also positively correlated with [^3^C*]_ss_, albeit weaker compared to [^1^O_2_*]_ss_, for the Hong Kong PM_2.5_ filter extracts.^16^

Beyond *E*_2_/*E*_3_, MAC_300_, and SUVA_254_, there remain significant gaps in our knowledge about how other optical parameters are related to [^1^O_2_*]_ss_, ΦO2*1 , [^3^C*]_ss_, and ΦC*3 for aqueous BrC. Despite their strengths likely varying by location and source, these relationships are key to identifying the physicochemical properties that reflect the quantity and quality of ^1^O_2_* and ^3^C* photosensitizers in aqueous BrC. Previous studies on aquatic chromophoric dissolved organic matter (CDOM) have demonstrated that absorbance and fluorescence parameters that correlate with CDOM physicochemical properties can be used to develop linear regression models to predict oxidant quantum yields.^24^ To date, this approach has not been applied to aqueous BrC. Since aquatic CDOM and aqueous BrC have different composition and physicochemical properties, it is currently unknown whether this modeling approach can be applied to model oxidant production in aqueous BrC. However, its successful implementation can potentially reduce the amount of time and labor spent on photochemical experiments performed to elucidate ^1^O_2_* and ^3^C* production by aqueous BrC.

Due to the chemical complexity of atmospheric BrC, many questions remain about the contributions of different photosensitizers to ^1^O_2_* and ^3^C* production. To date, there has only been one study that apportioned the contributions of different photosensitizers to oxidant production. By correlating the [^1^O_2_*]_ss_ for aqueous BrC extracts with organic aerosol factors identified by positive matrix factorization (PMF) analysis performed on the data sets concurrently measured by an aerosol mass spectrometer (AMS), Bolger et al. (2022) reported that photosensitizers originating from biomass burning and anthropogenic emissions were the strongest contributors to [^1^O_2_*]_ss_ for the San Vittore and Frauenfeld (Switzerland) PM_10_ filters, respectively.^10^ Nevertheless, concurrent ^1^O_2_* and ^3^C* and AMS data sets are scarce, necessitating other more accessible methods to apportion the contributions of different photosensitizers to ^1^O_2_* and ^3^C* production in aqueous BrC.

Excitation–Emission Matrix (EEM) fluorescence spectroscopy coupled with parallel factor (PARAFAC) analysis is commonly used to resolve complex EEM fluorescence spectra of atmospheric BrC into its dominant fluorescent components, which can then be used to understand their composition and sources.^25–30^ Petersen-Sonn et al. reported that different PARAFAC-resolved components were positively correlated with [^1^O_2_*]_ss_ and [^3^C*]_ss_, albeit at different strengths, for Grenoble PM_10_ filter extracts.^7^ While their results indicated that photosensitizers in these components contributed to ^1^O_2_* and ^3^C* production, their relative contributions are unknown without a source apportionment analysis. For a variety of BrC (e.g., coal and wood combustion, anthropogenic (ASOA) and biogenic secondary organic aerosols (BSOA)), Chen et al. used PARAFAC analysis to apportion the contributions of different fluorescent components to the decay of 2,4,6-trimethylphenol (TMP), a ^3^C* probe, induced by its reaction with ^3^C* species formed from the BrC.^31^ However, their analysis did not account for TMP degradation by ^1^O_2_*, which is needed for the accurate quantification of ^3^C* production. Nevertheless, results from Petersen-Sonn et al. and Chen et al. raise the possibility of using ^1^O_2_*, ^3^C*, and EEM fluorescence data sets combined with PARAFAC analysis to apportion the contributions of different photosensitizers to ^1^O_2_* and ^3^C* production.

In this study, we used ^1^O_2_* and ^3^C* and EEM fluorescence data sets combined with PARAFAC and non-negative matrix factorization (NMF) analyses to apportion the contributions of different photosensitizers to [^1^O_2_*]_ss_, [^3^C*]_ss_, ΦO2*1 , and ΦC*3 for aqueous BrC extracts from PM_2.5_ filters collected during a year-long study conducted in Hong Kong, a densely populated subtropical city located on the east of the Pearl River Delta in South China. In addition, we measured a variety of absorbance and fluorescence parameters for the aqueous BrC extracts, and investigated their correlations with the measured [^1^O_2_*]_ss_, [^3^C*]_ss_, ΦO2*1 , and ΦC*3 to identify key physicochemical properties that reflect the quantity and quality of ^1^O_2_* and ^3^C* photosensitizers. Based on these correlations, we developed linear regression models that can predict [^1^O_2_*]_ss_, [^3^C*]_ss_, ΦO2*1 , and ΦC*3 from absorbance and fluorescence parameters. This study demonstrates how measurable physicochemical properties of BrC could be used to apportion and understand the relative contributions of different photosensitizers to ^1^O_2_* and ^3^C* production in aqueous BrC, as well as develop predictive models on ^1^O_2_* and ^3^C* production.

## 2 Methods

### 2.1 PM_2.5_ Sampling and Extraction

Description about the sites, sample collection and extraction are detailed in our previous work.^16^ Briefly, PM_2.5_ samples were collected continuously on three 47 mm prebaked quartz filters for 72 h on every third day from December 2020 to December 2021 at three sites in Hong Kong. To minimize aging during storage, all PM_2.5_ filter samples were immediately wrapped in aluminum foil, sealed in airtight Ziplock bags, and stored in the dark at −20 °C until extraction. Filter samples were typically extracted within 1 week of collection. Each filter was extracted in 7 mL Milli-Q water. Extracts from three consecutive sampling periods (nine filters in 9 days) were subsequently aggregated to minimize daily variability. All extracts were stored at 4 °C in the dark for up to 6 months, from the date of extraction to the completion of the photochemical experiments. Only 28 out of the total 34 aggregated extracts were used for this study (Table S1). One of the 6 excluded aggregated extracts had far-outlier steady-state concentration and quantum yield values,^16^ while the remaining 5 excluded aggregated extracts had insufficient liquid volume for absorbance and fluorescence measurements. The concentrations of water-soluble organic carbon (WSOC) of the extracts were measured using a total organic carbon (TOC) analyzer (Shimadzu TOC-VCSH), which was calibrated using potassium hydrogen phthalate and sucrose standards.

### 2.2 Absorbance and Fluorescence Measurements

Absorption spectra of filter extracts were measured using a UV–vis spectrophotometer (Shimadzu UV-3600) and a pair of 1 cm quartz cuvettes. Absorption spectra were recorded in the wavelength range of 200 to 800 nm and corrected for the baseline drift.^21^ The absorption spectra were subsequently used to obtain 11 absorbance parameters (α_254,_ α_300,_ α_365_, *R*_abs_, MAC_300_, SUVA_254_, SUVA_365_, *E*_2_/*E*_3_, *S*_275–295_, *S*_350–400_, and *S*_R_). EEM fluorescence spectra were measured using a spectrofluorometer (Horiba Fluormax-4). The excitation wavelengths (λ_ex_) were set from 200 to 450 at 10 nm intervals, whereas the emission wavelengths (λ_em_) were set from 250 to 550 at 5 nm intervals. The scanning was operated in the ratio mode (*S*/*R*) with a slit width of 5 nm.

The measured EEM fluorescence spectra were corrected using previously reported methods: Rayleigh and Raman scattering elimination, inner filter correction, blank subtraction, and calibration (normalization to the Raman scattering band of water, Raman unit).^28^ The EEM fluorescence spectra were corrected and analyzed using PARAFAC in R Studio (package *staRdom* v1.1.25) to decompose the spectra into separate fluorescent components and quantify each component’s contribution to the total fluorescence. The optimal number of components was determined based on the core consistency diagnostic and visual inspection of residuals.^32^ After comparing the results of 3- to 6-component models, the 3-component model was chosen. The components were identified based on previous studies of the fluorescence characteristics of aqueous BrC in Guangzhou,^30^ another densely populated city located in South China that shares several similar air quality characteristics as Hong Kong. The EEM fluorescence measurements were used to obtain 19 fluorescence parameters (FI, BIX, HIX, Peak A, Peak C, Peak M, Peak B, Peak T, Peak A:T, Peak C:T, Peak C:A, Peak C:M, Comp. 1, Comp. 2, Comp. 3, Comp. 123, Rel. Comp. 1, Rel. Comp. 2, and Rel. Comp. 3). Descriptions and calculations of the absorbance and fluorescence parameters are detailed in Table S2.

### Quantification of [^1^O_2_*]_ss_, ΦO2*1 , [^3^C*]_ss_, and ΦC*3

2.3

The [^1^O_2_*]_ss_, ΦO2*1 , [^3^C*]_ss_, and ΦC*3 values for the 28 extracts have been reported in our previous work.^16^ Details of the photochemical experiments performed to quantify [^1^O_2_*]_ss_, [^3^C*]_ss_, ΦO2*1 , and ΦC*3 can be found in our previous work.^16^ Briefly, photochemical experiments were conducted in a Rayonet photoreactor (RPR-200; Southern New England Ultraviolet Co.) equipped with 12 UVA lamps (RPR-3500A; Southern New England Ultraviolet Co.). The spectral irradiance in the photoreactor is shown in Figure S1. The production of ^1^O_2_* and ^3^C* were quantified in separate experiments using probes furfuryl alcohol (FFA, 10 μM) and syringol (SYR, 10 μM), respectively.^13,33^ The decays of FFA and SYR were measured using an ultrahigh-pressure liquid chromatography system coupled to a photodiode array detector (UPLC-PDA; Waters ACQUITY H-Class). Reaction times were carefully chosen to minimize photobleaching and secondary reactions. As shown in our previous work,^16^ the initial decays of FFA and SYR followed pseudo first-order kinetics. The kinetic solvent isotope effect (KSIE) was used to account for FFA degradation by oxidants other than ^1^O_2_* during photochemical experiments.^34^ This involved comparing the decay of FFA in pure water (H_2_O) to that in heavy water (D_2_O). Due to the chemical complexity of the ^3^C* pool and the use of a single ^3^C* probe, only a subset of ^3^C* species that can oxidize SYR via electron transfer reactions were quantified in this study, whereas ^3^C* species that react with organic species via energy transfer reactions were not.^35,36^ We used the [^1^O_2_*]_ss_ and ΦO2*1 data sets obtained from our previous study.^16^ However, we corrected the [^3^C*]_ss_ and ΦC*3 data sets obtained from the aforementioned study for the inhibitory effect of WSOC on SYR oxidation by ^3^C* (Section S1).^17,23,37^ These corrections resulted in increases of [^3^C*]_ss_ and ΦC*3 by, on average, 21% compared to the previously reported data set (sample-wise from 6 to 39%). The [^1^O_2_*]_ss_, [^3^C*]_ss_, ΦO2*1 , and ΦC*3 data sets used in this study are shown in Table S3, while their seasonal variations are shown in Figure S2.

### 2.4 Statistical Analyses

NMF analysis was performed using MATLAB (version R2024a) to apportion the contributions of PARAFAC-resolved components to [^1^O_2_*]_ss_, [^3^C*]_ss_, ΦO2*1 , and ΦC*3 . NMF, a dimensionality reduction technique that decomposes a non-negative data matrix into two lower-rank non-negative matrices to reveal underlying patterns in complex data sets, has previously been used to apportion the contributions of PARAFAC-resolved fluorescent components to PMF-resolved AMS organic aerosol factors and oxidative potential of organic aerosols.^26,38^ The principle of NMF analysis is described by the following equation^39,40^

1 $$V_{28\times 4}=W_{28\times n}\times H_{n\times 4}+D$$

where *V*_28×4_ is the product matrix containing [^1^O_2_*]_ss_, [^3^C*]_ss_, ΦO2*1 , and ΦC*3 for the 28 aggregated extracts, *W*_28×*n*_ is the basic matrix containing the extract-specific intensities of each PARAFAC-resolved component, *n* is the number of PARAFAC-resolved components, *H*_*n*×4_ is the coefficient matrix representing the weights or contributions of each basis component to [^1^O_2_*]_ss_, [^3^C*]_ss_, ΦO2*1 , and ΦC*3 , and *D* is residual matrix. The NMF analysis was ran 10,000 times to derive *H*_*n*×4_, which was used to determine the relative contributions of each component. To ensure good performance of the NMF analysis, the data set was scaled to the range of 0 to 1 without changing the distribution of data set.

Spearman correlation analysis was performed using R (*linkET* package) to investigate the relationships between measurements of the 30 absorbance and fluorescence parameters, WSOC concentration, [^1^O_2_*]_ss_, [^3^C*]_ss_, ΦO2*1 , and ΦC*3 . The absorbance and fluorescence parameters were selected based on their usage in previous studies to describe specific physicochemical properties (e.g., aromaticity, molecular size) and chromophoric organic matter classes (e.g., sources and oxidation levels).^1,29,41^ The nonparametric Spearman rank correlation coefficient (*r*) was used to identify monotonic associations, minimizing the influence of outliers and nonlinear trends. Correlations were also evaluated for statistical significance with *p* < 0.05 indicating that the correlation is statistically significant.

A two-step modeling approach was employed to develop predictive multiple linear regression (MLR) models for [^1^O_2_*]_ss_, [^3^C*]_ss_, ΦO2*1 , and ΦC*3 based on measured absorbance and fluorescence parameters and WSOC concentrations. Using a procedure similar to Wasswa et al. and Li et al.,^24,42^ orthogonal partial least-squares (OPLS) analysis was first performed using R to identify the most relevant predictor variables. OPLS, which is a supervised statistical analysis technique, can model the relationships between response variables (i.e., [^1^O_2_*]_ss_, [^3^C*]_ss_, ΦO2*1 , and ΦC*3 ) and collinear predictor variables (i.e., absorbance and fluorescence parameters and WSOC concentrations).^43^ OPLS is particularly suited for small sample sizes (such as our study which has 28 samples) since it improves model interpretability by separating systematic variation in the predictor variables into predictive variation (correlated to the responses) and orthogonal variation (uncorrelated to the responses).^44^ Each model was optimized to maximize both the explained variance in the response variables and the model’s predictive capability, as determined by cross-validation. The relative importance of each predictor variable was assessed using the Variable Importance in Projection (VIP) score,^43^ where a VIP score >1.0 indicates a variable of high influence.^45^ Parameters with a VIP score greater than 1.0 were subsequently selected as predictors for stepwise MLR, thus ensuring that only the most influential parameters were retained for further modeling. Stepwise MLR models for [^1^O_2_*]_ss_, ΦO2*1 , [^3^C*]_ss_, and ΦC*3 were developed using the selected optical variable predictors in IBM SPSS Statistics (version 29.0.1.0).

## 3 Results and Discussion

### 3.1 PARAFAC-Resolved Fluorescent Components

PARAFAC analysis was performed on EEM fluorescence spectra of the aqueous extracts of PM_2.5_ samples to resolve the BrC into their dominant components. Figure 1 shows the three components resolved by PARAFAC analysis: Comp. 1 (λ_ex_/λ_em_ = 240, 310/410 nm), Comp. 2 (λ_ex_/λ_em_ = 260, 350/460 nm), and Comp. 3 (λ_ex_/λ_em_ = 240, 280/355 nm). Comp. 2 had longer excitation and emission wavelengths than Comp. 1, indicating that Comp. 2 is comprised of chromophores with more aromatic structures and/or unsaturated bonds and are more oxygenated compared to Comp. 1.^28^ In contrast, Comp. 3 had the shortest excitation and emission wavelengths out of the three components, indicating that it is comprised of chromophores with smaller molecular sizes compared to Comp. 1 and Comp. 2.^28^ The EEM fluorescence spectra of the three components agreed with those previously reported for aqueous extracts of PM_2.5_ from Guangzhou, a similar megacity in the region.^30^

**Figure 1:**
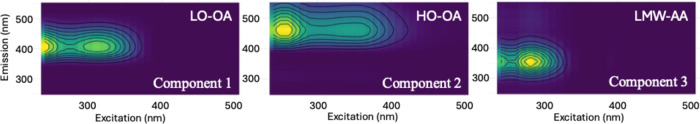
Three fluorescent components derived from the optimal PARAFAC modeling of the aqueous extracts of PM_2.5_ samples.

Based on comparisons of the EEM fluorescence spectra of the three components to those from previous studies,^28,30^ Comp. 1 could be attributed to less oxygenated chromophoric BSOA and ASOA from biogenic and anthropogenic volatile organic compounds (VOCs), respectively. Previous studies reported that Comp. 2 could be attributed to highly oxygenated chromophoric organic aerosols from atmospheric aging due to Comp. 2 having a high oxygen content and O/C ratio.^25,27^ However, the EEM fluorescence spectra of oxygenated HULIS from atmospheric aging, high-ring-number polyaromatic hydrocarbons (PAHs) and their derivatives (e.g., fluoranthene, benzo­[*b*]­fluoranthene, benzo­[*a*]­pyrene, indeno­[1,2,3-cd]­pyrene), and nitrogen-containing heterocyclic compounds (NCHCs) (e.g., imidazole-2-formaldehyde) also overlap with that of Comp. 2,^25,30,46–49^ suggesting that these classes of compounds may also contribute to Comp. 2. Comp. 3 could be attributed to low molecular weight aromatic acids (e.g., 3,5-dihydroxybenzoic acid, 2-napthalenecarboxylic acid), PAHs (e.g., naphthalene, phenanthrene, anthraquinone), and phenolic compounds (e.g., catechol, hydroquinone, 2-methoxyphenol).^30,50^ It is important to note that the aforementioned compounds serve as inferred chromophoric structures based on optical data, not as direct identification of their presence in the aqueous PM_2.5_ extracts. For simplicity, we hereafter refer to Comp. 1, Comp. 2, and Comp. 3 as less oxygenated organic aerosols (LO-OA), highly oxygenated organic aerosols (HO-OA), and low molecular weight aromatic acids (LMW-AA) components, respectively (Figure 1).

The LO-OA component had the highest relative abundance, accounting for, on average, 50 ± 5% of the total fluorescence intensity (Figure S3). The HO-OA and LMW-AA components comprised, on average, 28 ± 3% and 22 ± 7% of the total fluorescence intensity, respectively (Figure S3). The high abundance of the LO-OA component could be attributed to SOA from both local and regional biogenic and anthropogenic VOC emissions, which are prevalent under Hong Kong’s subtropical conditions and its mix of urban and vegetated landscapes.^51–53^ Figure 2a–2c show that the seasonal variations of the absolute fluorescence intensities (*F*_max_) of the three components were significant (*p* < 0.05). *F*_max_ for the three components were the highest in winter and the lowest in summer. These seasonal variations could be driven by the East Asian monsoon system, with polluted northern continental air masses leading to high concentrations of PM_2.5_ and BrC in fall and winter and clean southern marine air masses leading to low concentrations of PM_2.5_ and BrC in summer.^54,55^ In contrast, significant seasonal variation in relative abundance was observed only for the HO-OA component (*p* < 0.05), while the LO-OA and LMW-AA components showed no significant seasonal changes (Figure 2d–f).The higher relative abundance of the HO-OA component in winter and fall highlights the significant import of aged organic aerosols transported from northern polluted continental areas.

**Figure 2:**
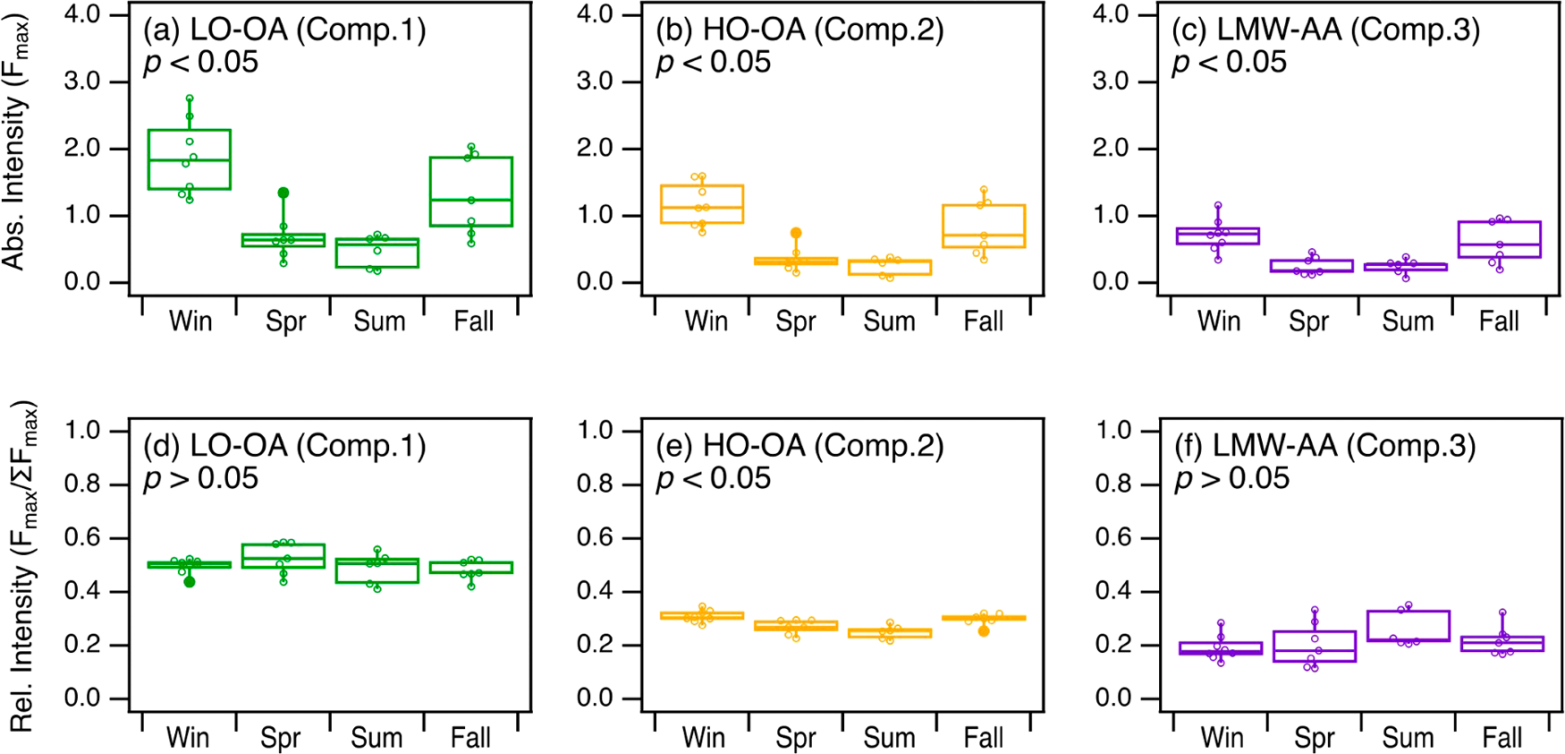
Seasonal variations in the (a–c) absolute intensities and (d–f) relative intensities of the PARAFAC-resolved components. The relative intensities (*F*_max_/Σ*F*_max_) were calculated by dividing the absolute intensity of each component (*F*_max_) by the sum of intensities of the three components (*∑F*_max_).^56^ For panels (a)–(f), the whiskers denote the 10th and 90th percentile values, the boxes denote the 25th and 75th percentile values, and the midlines denote the median values. Data points are shown as open symbols. Statistical significances in the seasonal variations were assessed using one-way ANOVA, with *p* < 0.05 indicating significant difference and *p* > 0.05 indicating no significant difference.

### 3.2 Contributions of Fluorescent Components to ^1^O_2_* and ^3^C* Production in Winter vs Summer

Our previous study showed that Hong Kong’s seasonal meteorological conditions and PM_2.5_ sources resulted in significant differences in the composition and properties of summer (June to August) vs winter (December to February) BrC, which led to discernible differences in [^1^O_2_*]_ss_ and ΦO2*1 (Figure 3a,b).^16^ These differences were not as apparent when compared to spring (March to May) and fall (September to November) (Figure S2) since the spring and fall seasons were marked by fluctuating air masses and meteorological conditions. Thus, to gain insights into discernible seasonal contributions of ^1^O_2_* and ^3^C* photosensitizers, we used NMF to investigate the contributions of PARAFAC-resolved components to [^1^O_2_*]_ss_, [^3^C*]_ss_, ΦO2*1 , and ΦC*3 for winter vs summer. The reconstructed matrix, *V*_reconstructed_, successfully represented the actual product matrix, *V*, with a small residual matrix, *D* (Figure S4).

**Figure 3:**
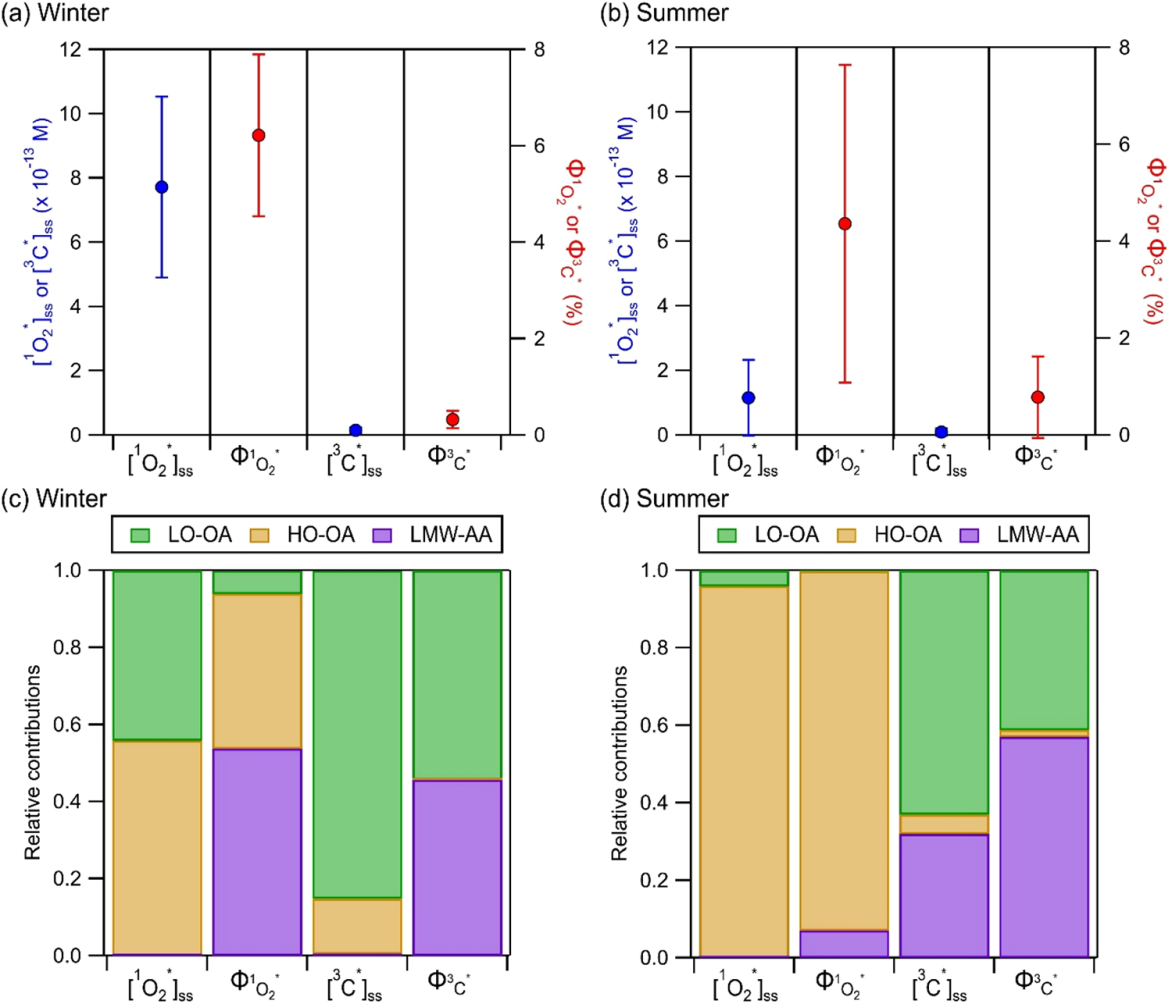
Average [^1^O_2_*]_ss_, ΦO2*1 , [^3^C*]_ss_, and ΦC*3 for the (a) winter vs (b) summer extracts. Error bars denote one standard deviation. Relative contributions of the PARAFAC-resolved components to [^1^O_2_*]_ss_, ΦO2*1 , [^3^C*]_ss_, and ΦC*3 in (c) winter vs (d) summer extracts as determined by NMF analysis.

The NMF results shown in Figure 3c,3d revealed that the LO-OA component had the highest relative contributions to [^3^C*]_ss_ in winter (85%) and summer (63%), during which it comprised majority of the total fluorescence intensity (50 ± 3% and 49 ± 6%, respectively). The HO-OA component had the highest relative contributions to [^1^O_2_*]_ss_ in winter (56%) and summer (93%), despite it comprising, on average, 31 ± 2% and 25 ± 3% of the total fluorescence intensity, respectively. Differences in the contributions of fluorescent components to [^1^O_2_*]_ss_ vs [^3^C*]_ss_ could be due to ^3^C* species having different efficiencies at transferring energy to O_2_ to form ^1^O_2_*. This is demonstrated by the range in ΦO2*1 for model ^1^O_2_* photosensitizers typically used to generate ^1^O_2_* in laboratory oxidation studies (e.g., perinaphthenone ( ΦO2*1 = 98%), rose bengal ( ΦO2*1 = 75%), and methylene blue ( ΦO2*1 = 37 to 56%)).^21,35^ Additionally, even though ^3^C* are precursors for ^1^O_2_* formation, not all ^3^C* photosensitizers are ^1^O_2_* photosensitizers. For example, although SOA formed from the ^•^OH photooxidation of α-pinene contained ^3^C* photosensitizers,^31^ they form little to no ^1^O_2_*.^8^ Furthermore, only a subset of ^3^C* species that can oxidize syringol (the ^3^C* probe used) were captured in our photochemical experiments.

The NMF results for [^1^O_2_*]_ss_ and [^3^C*]_ss_ were noticeably different from those for ΦO2*1 and ΦC*3 . This could be explained by differences in the concentrations (i.e., quantity) and photosensitization abilities (i.e., the quality) of BrC chromophores in the three components. If BrC chromophores in the component are weak ^1^O_2_* (or ^3^C*) photosensitizers, they can be minor contributors to [^1^O_2_*]_ss_ (or [^3^C*]_ss_) even if they are present in large concentrations. Conversely, the ^1^O_2_* (or ^3^C*) photosensitization efficiencies of BrC chromophores in the component need to be high enough to offset their low concentrations in order for them to be significant contributors to [^1^O_2_*]_ss_ (or [^3^C*]_ss_). The NMF results suggested that chromophores in the LO-OA component were substantially weaker ^1^O_2_* photosensitizers compared to chromophores in the HO-OA component since the LO-OA component was not the main contributor to [^1^O_2_*]_ss_ in winter and summer despite its large abundances (Figure 1b,e). The dominant contribution of the HO-OA component to ΦO2*1 and [^1^O_2_*]_ss_ in summer (96% and 93%, respectively) suggested that the ^1^O_2_* photosensitization efficiencies of its chromophores were high enough to offset its low abundance (Figure 1c,1f). In contrast, despite the LMW-AA component being the main contributor to ΦO2*1 in winter (54%), it was a negligible contributor to [^1^O_2_*]_ss_ due to its low abundance in winter (Figure 2c,2f). Although the LO-OA and LMW-AA components were major contributors to winter and summer ΦC*3 , the LO-OA component was the highest contributor to [^3^C*]_ss_ due to its substantially higher abundance compared to the LMW-AA component. The substantial contributions of the LMW-AA component to winter and summer ΦC*3 and to winter ΦO2*1 suggested that its chromophores are strong ^1^O_2_* and ^3^C* photosensitizers. This could be attributed to the small molecular sizes of its chromophores, which consist of chromophoric small molecular weight aromatic acids and phenolic compounds.^30,50^ Their small molecular sizes minimize charge-transfer interactions, thus reducing the occurrence of electron quenching processes that disrupt photochemical processes including ^3^C* and ^1^O_2_* production.^57^ Overall, the NMF results underscored the importance of considering both the quantity and quality of BrC chromophores in the production of ^1^O_2_* and ^3^C*.

Based on the NMF results, LO-OA chromophores were the primary contributors to winter and summer ^3^C* production. This aligns with work by Chen et al., who demonstrated the strong ^3^C* photosensitization abilities of various laboratory-generated BSOA and ASOA.^31^ Conversely, HO-OA chromophores were the primary contributors to winter and summer ^1^O_2_* production. HO-OA chromophores contain not just highly oxygenated organic aerosols, but also oxygenated HULIS, high-ring-number PAHs and their derivatives, and NCHCs (Section 3.1).^25,30,46–49^ Some of the aforementioned organic aerosol components are known ^1^O_2_* photosensitizers. In particular, Manfrin et al. who showed that SOA produced from ^•^OH photooxidation of aromatic VOCs (toluene, biphenyl, naphthalene, and 1,8-dimethylnaphthalene) had ΦO2*1 up to 3%.^8^ Although the SOA contained both oxygenated aromatic compounds and oxygenated ring-opening compounds, the authors reported that oxygenated aromatic compounds were the main ^1^O_2_* photosensitizers, with an increase in aromatic content leading to higher light absorption rates and [^1^O_2_*]_ss_. Additionally, Felber et al. (2020) showed that the excited states of some NCHCs (e.g., imidazole-2-carboxaldehyde) react easily with O_2_ to produce ^1^O_2_*.^58^ Collectively, these results indicate that the oxidation of biogenic and anthropogenic VOCs and atmospheric aging processes are major sources of ^3^C* and ^1^O_2_* photosensitizers in Hong Kong’s PM_2.5_.

### Correlations between Physicochemical Properties, [^1^O_2_*]_ss_, ΦO2*1 , [^3^C*]_ss_, and ΦC*3

3.3

Correlation analysis revealed several significant associations among the absorbance and fluorescence parameters and WSOC concentration of aqueous BrC extracts (Figure 4). Since these absorbance and fluorescence parameters are known proxies for specific physicochemical properties of chromophoric organic matter (Table S2), their correlations provide insights into the underlying characteristics of the BrC. Strong positive correlations (*r* > 0.5, *p <* 0.05) were observed between WSOC concentration and UV absorbance coefficients, α_254,_ α_300,_ α_365_, and MAC_300_, indicating that higher concentrations of WSOC led to stronger absorption. Furthermore, the intensities of PARAFAC-resolved components and fluorescence peaks that denote HULIS (Peak A), fulvic acid (Peak C), marine-derived HULIS (Peak M), tyrosine-like organic matter (Peak B), and tryptophan-like organic matter (Peak T) were strongly positively correlated with α_254,_ α_300,_ α_365_, and MAC_300_ (*r* > 0.5, *p <* 0.05), highlighting the diverse composition and sources of BrC. The WSOC-normalized absorbance parameters, SUVA_254_ and SUVA_365_, were strongly negatively correlated with *E*_2_/*E*_3_, spectral slopes *S*_275–295_ and *S*_350–400_, and the fluorescence index (FI) (*r* < *–*0.5, *p <* 0.05), indicating that BrC species with higher aromaticity and/or larger molecular sizes were associated with stronger absorption. The biological index (BIX) showed a correlation pattern similar to, albeit weaker than, FI. BIX correlated negatively with SUVA_254_ and SUVA_365_ (*r* < *–*0.5, *p <* 0.05), but positively with *E*_2_/*E*_3_, *S*_275–295_, and *S*_350–400_ (*r* > 0.5, *p <* 0.05). Although BIX is a parameter that is commonly used to assess the contributions of microbial activities in aquatic environments, it has been shown to be negatively correlated with the aromaticity and molecular weight of organic matter.^59^ Thus, the observed correlations between BIX and SUVA_254_, SUVA_365_, *E*_2_/*E*_3_, *S*_275–295_, and *S*_350–400_ are consistent with BrC species with higher aromaticity and/or larger molecular sizes being associated with stronger absorption.

**Figure 4:**
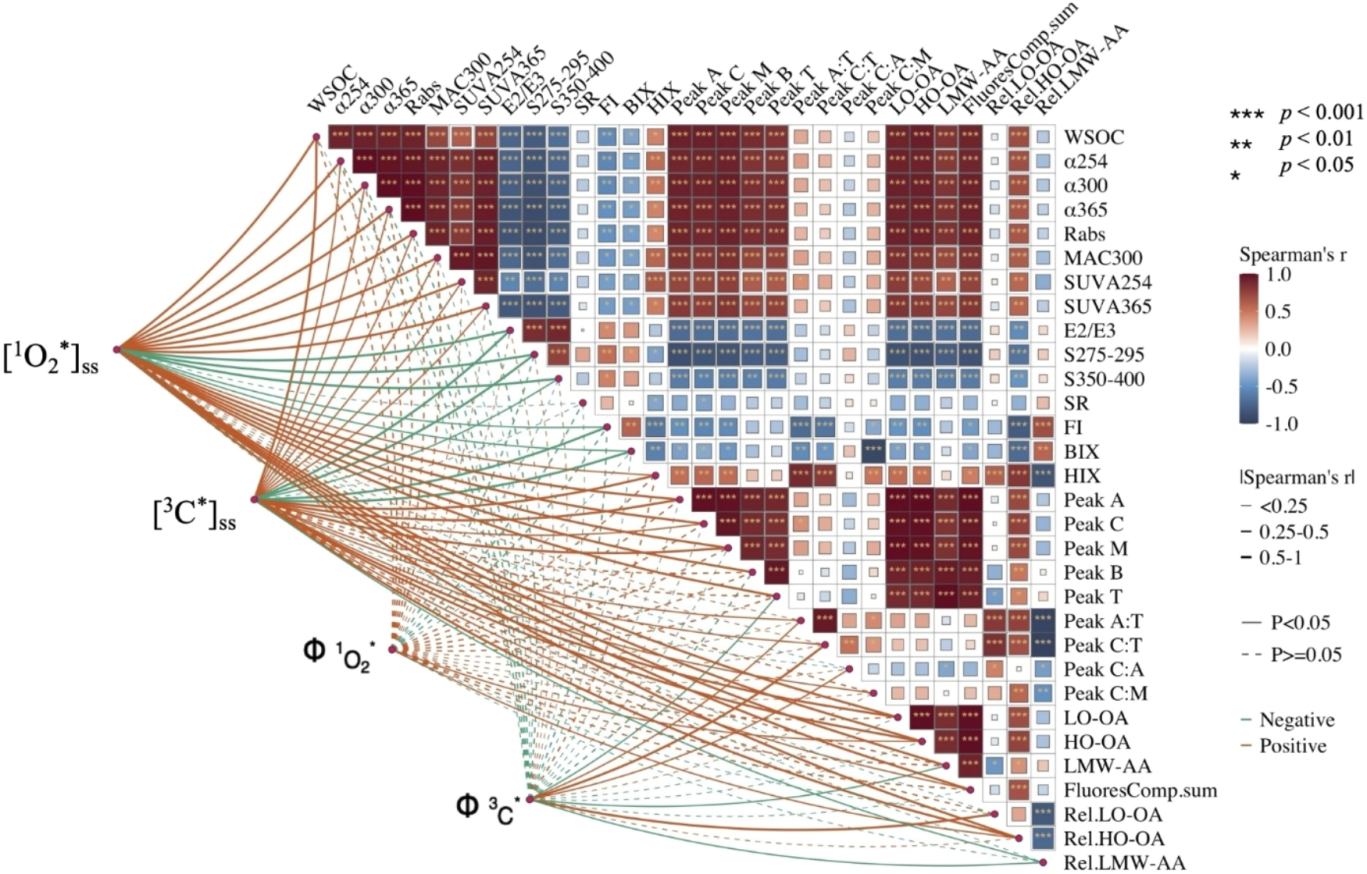
Spearman rank correlations between the BrC absorbance and fluorescence parameters, WSOC concentration, [^1^O_2_*]_ss_, [^3^C*]_ss_, ΦO2*1 , and ΦC*3 . Spearman’s rank correlation coefficients (*r*) values and statistical significance test results are shown in Table S4. The thickness of the lines connecting [^1^O_2_*]_ss_/ ΦO2*1 /[^3^C*]_ss_/ ΦC*3 to absorbance and fluorescence parameters and WSOC concentration corresponds to the magnitude of absolute value of *r* (thicker: stronger, thinner: weaker), with solid lines denoting statistically significant correlations (*p* < 0.05) and dashed lines indicating statistically insignificant correlations (*p* ≥ 0.05). Red lines denote positive correlations (*r* > 0), whereas green lines denote negative correlations (*r* < 0). The heatmap also depicts the *r* values among the absorbance and fluorescence parameters and WSOC concentrations, with color intensity reflecting correlation strength (darker red and blue indicate stronger positive correlations (*r* > 0.5) and stronger negative correlations (*r* < *–*0.5), respectively).

[^1^O_2_*]_ss_ and [^3^C*]_ss_ had significant correlations (*p* < 0.05) with a large number of absorbance and fluorescence parameters in addition to their positive correlations with MAC_300_ and SUVA_254_ reported in our previous study (Figure 4 and Table S4). *E*_2_/*E*_3_, *S*_275–295_, *S*_350–400_, FI, and BIX had significant negative correlations with both [^1^O_2_*]_ss_ and [^3^C*]_ss_ (*p* < 0.05), indicating that the concentrations of ^1^O_2_* and ^3^C* produced were closely associated with the presence of large aromatic compounds. The intensities of the LO-OA component, HO-OA component, Peak A (denotes HULIS), Peak C (denotes fulvic acid), and Peak M (denotes marine-derived HULIS) were also strongly positively correlated with both [^1^O_2_*]_ss_ and [^3^C*]_ss_, indicating that a large fraction of fluorescent species contributed to ^1^O_2_* and ^3^C* production. ΦO2*1 and ΦC*3 had significant correlations (*p* < 0.05) with substantially fewer absorbance and fluorescence parameters compared to [^1^O_2_*]_ss_ and [^3^C*]_ss_. Although positive correlations were observed between ΦO2*1 and E_2_/E_3_ for aqueous BrC extracts of PM filters from Davis and Grenoble and Toronto road dust samples,^7,18,23^ both ΦO2*1 and ΦC*3 had no to weak correlations with *E*_2_/*E*_3_ in this study. This indicated that the strengths of correlations between ΦO2*1 (or ΦC*3 ) and molecular weight of chromophores (i.e., *E*_2_/*E*_3_) depend on location and BrC sources. Notably, the relative intensities of the HO-OA and LO-OA components were positively correlated with ΦO2*1 and ΦC*3 , respectively (*p* < 0.05). This is consistent with the NMF results (Section 3.2) which showed that the HO-OA and LO-OA components contained efficient ^1^O_2_* and ^3^C* photosensitizers, respectively, and thus were major contributors to ΦO2*1 and ΦC*3 , respectively.

### Predicting [^1^O_2_*]_ss_, [^3^C*]_ss_, ΦO2*1 , and ΦC*3 from Measurable Physicochemical Properties

3.4

The observed correlations between [^1^O_2_*]_ss_ and [^3^C*]_ss_ (or ΦO2*1 and ΦC*3 ) and the absorbance and fluorescence parameters of BrC (Section 3.3) suggest that these parameters could be used to develop predictive MLR models for [^1^O_2_*]_ss_ and [^3^C*]_ss_ (or ΦO2*1 and ΦC*3 ). OPLS analysis was first applied to address multicollinearity among the 31 measured parameters,^43^ selecting only those with a VIP score greater than 1.0 to prevent overfitting and prioritize optically relevant predictors (Section 2.4).^45^ This process resulted in 18 and 20 parameters being selected as predictive variables for [^1^O_2_*]_ss_ and [^3^C*]_ss_ predictions, respectively, and 15 and 14 variables selected as predictive variables for ΦO2*1 and ΦC*3 predictions, respectively (Figure S5).

Stepwise MLR models for [^1^O_2_*]_ss_, [^3^C*]_ss_, ΦO2*1 , and ΦC*3 were subsequently developed using the parameters prioritized by the OPLS analysis. In each case, stepwise MLR identified a single, statistically significant parameter that adequately explained the variance: α_254_ for [^1^O_2_*]_ss_, BIX for [^3^C*]_ss_, Rel. Comp. 2 for ΦO2*1 , and Comp. 3 for ΦC*3 . The inclusion of additional parameters did not significantly improve the MLR fits. This underscored the dominant influence of each selected parameter. The predictive performance of each MLR model was evaluated by fitting a linear regression to the predicted vs measured values with the intercepts set to 0 (Figure 5). The resulting slopes ranged from 0.77 to 0.95, with coefficient of determination (*r^2^*) values between 0.69 and 0.95. Nearly all of the predicted values fell within the 95% prediction bands. These fits indicated overall good agreement between predicted steady-state concentrations and quantum yields and their corresponding measured values. However, the slopes of the fits were consistently below unity. This trend, combined with the noticeable overprediction of ΦO2*1 and ΦC*3 at lower measured values, suggests that the measured absorbance and fluorescence parameters did not fully capture the entire complex pool of photosensitizing BrC chromophores present in the samples. Despite these deviations, the results successfully demonstrate the feasibility of using a combined OPLS-MLR modeling approach and the standard absorbance and fluorescence parameters to predict the production of ^1^O_2_* and ^3^C* from BrC.

**Figure 5:**
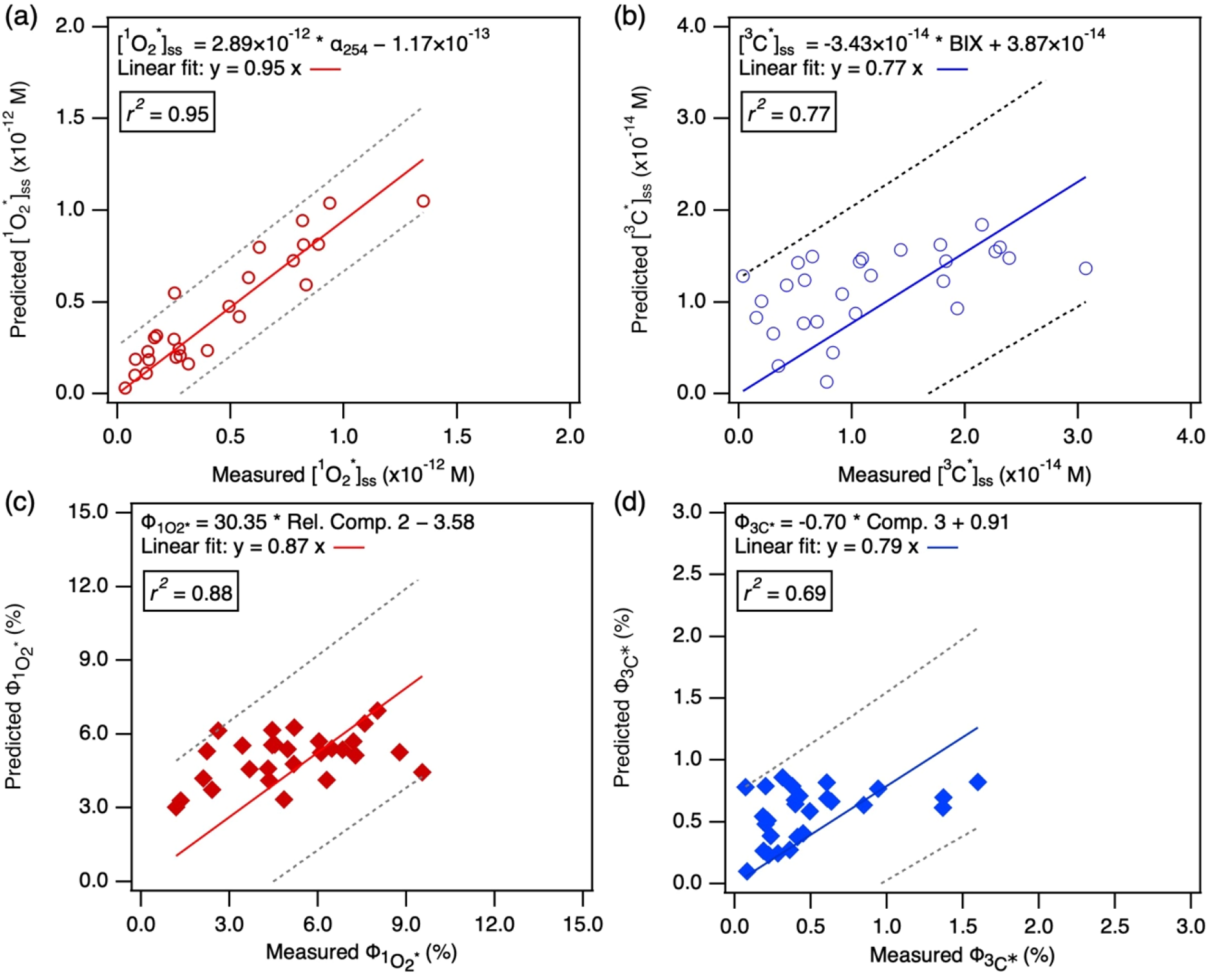
Predicted vs measured (a) [^1^O_2_*]_ss_, (b) [^3^C*]_ss_, (c) ΦO2*1 , and (d) ΦC*3 values from combined OPLS and MLR analyses. The red and blue lines are linear fits to the data points for ^1^O_2_* and ^3^C*, respectively, with the intercepts set to 0. The goodness of the fits indicated by *r* ^2^ are shown in boxes. The dashed lines indicate the 95% prediction bands for the linear fit. These bands define the interval within which we would expect 95% of new data points to lie in if the same model holds. The equation of the MLR model used for prediction is shown on top of each panel.

Better agreement between measured and predicted values was achieved for [^1^O_2_*]_ss_ and [^3^C*]_ss_ compared to ΦO2*1 and ΦC*3 . The most effective predictors were α_254_ for [^1^O_2_*]_ss_ and BIX for [^3^C*]_ss_ (Figure 5a,5b). This finding is consistent with the previously established significant correlations for α_254_ vs [^1^O_2_*]_ss_ (*r* = 0.90) and BIX vs [^3^C*]_ss_ (*r* = −0.59) (Table S4). The MLR models implied that [^1^O_2_*]_ss_ was mainly controlled by the total concentration of chromophoric organic matter (as represented by α_254_), whereas [^3^C*]_ss_ was mainly governed by the concentration of chromophoric high molecular weight, aromatic organic compounds (as inferred from BIX). In contrast, ΦO2*1 and ΦC*3 were best predicted by composition-specific fluorescence parameters Rel. HO-OA and the LMW-AA component, respectively, This finding is consistent with the previously established significant correlations for Rel. HO-OA vs ΦO2*1 (*r* = 0.39) and LMW-AA vs ΦC*3 (*r* = −0.41) shown in Table S4, and is further supported by our NMF analyses which identified the HO-OA and LMW-AA components as significant contributors to ΦO2*1 and ΦC*3 , respectively (Section 3.2). Additionally, the MLR models revealed a key distinction between the main factors that control [^1^O_2_*]_ss_ and [^3^C*]_ss_ vs ΦO2*1 and ΦC*3 . [^1^O_2_*]_ss_ and [^3^C*]_ss_ were dependent on parameters that reflected the quantities of BrC chromophores, whereas the ΦO2*1 and ΦC*3 were influenced by the specific types (i.e., quality) of BrC chromophores present.

## 4 Atmospheric Implications

In this study, we demonstrated how the contributions of different photosensitizers to [^1^O_2_*]_ss_, [^3^C*]_ss_, ΦO2*1 , and ΦC*3 can be apportioned using ^1^O_2_* and ^3^C* and EEM fluorescence data sets combined with PARAFAC and NMF analyses. This analysis reveal new insights into the composition and physicochemical properties of key photosensitizers that contribute to aqueous ^1^O_2_* and ^3^C* production. In addition, we showed how a combined OPLS-MLR modeling approach can be employed to develop predictive MLR models for [^1^O_2_*]_ss_, [^3^C*]_ss_, ΦO2*1 , and ΦC*3 by leveraging correlations between their measured levels and absorbance and fluorescence parameters that serve as proxies for specific physicochemical properties of BrC. The MLR model-predicted values showed overall good agreement with their measured values, thus highlighting the feasibility of using standard absorbance and fluorescence parameters to predict ^1^O_2_* and ^3^C* production from aqueous BrC. HO-OA chromophores were found to be the main contributors to winter and summer ^1^O_2_* production by aqueous BrC in Hong Kong. In contrast, LO-OA chromophores were the main contributors to winter and summer ^3^C* production. The divergence between the NMF results for [^1^O_2_*]_ss_ and [^3^C*]_ss_ vs ΦO2*1 and ΦC*3 underscored that ^1^O_2_* and ^3^C* production depended on both the concentrations and intrinsic photosensitization efficiencies of their photosensitizers. Additionally, the MLR models revealed that [^1^O_2_*]_ss_ and [^3^C*]_ss_ depended on parameters that reflected the quantities of BrC chromophores, whereas ΦO2*1 and ΦC*3 were influenced by the specific types (i.e., quality) of BrC chromophores present.

The significance of our results lies foremost in our demonstration that easily measurable BrC physicochemical properties—derived from routine absorbance and fluorescence measurements—can be used to both apportion photosensitizer contributions and predict ^1^O_2_* and ^3^C* production via empirical modeling. By integrating PARAFAC, NMF, and OPLS-MLR, we establish a framework for reanalyzing concurrent data sets of ^1^O_2_*, ^3^C*, absorbance, and fluorescence measurements to evaluate ^1^O_2_* and ^3^C* production. In particular, the OPLS-MLR modeling approach will be useful for evaluating the atmospheric significance of aqueous ^1^O_2_* and ^3^C* production since it allows for high spatial-temporal resolution analysis in field studies with large ambient sample sets. We anticipate this approach will be widely applicable for identifying the key drivers of ^1^O_2_* and ^3^C* production (e.g., BrC sources, specific physicochemical properties) in other locations.

There are some caveats that should be noted. First, our probe-based photochemical experiments, while consistent with established methodologies for assessing ^1^O_2_* and ^3^C* production in ambient aerosols,^7,8,10,13–19^ determined [^1^O_2_*]_ss_, [^3^C*]_ss_, ΦO2*1 , and ΦC*3 under dilute conditions akin to ambient cloud and fog. These conditions do not fully represent concentrated ambient aerosols, where higher BrC concentrations (and those of other organic and inorganic compounds) could alter absorption behavior and reaction kinetics.^14,17,60,61^ Additionally, photosensitization occurred in bulk solution, which may not accurately replicate the reaction kinetics and absorption in actual atmospheric aqueous droplets and aerosols.^62,63^ Previous studies showed that [^1^O_2_*]_ss_ and [^3^C*]_ss_ increase with BrC concentration in extracts of ambient aerosols, albeit nonlinearly.^14,17,23^ Future work should consider developing methods for quantifying [^1^O_2_*]_ss_, [^3^C*]_ss_, ΦO2*1 , and ΦC*3 in airborne aerosols and aqueous droplets. Second, the absolute outputs of the MLR models (e.g., coefficient slopes) will depend on the specifics of the experimental setup since [^1^O_2_*]_ss_, [^3^C*]_ss_, ΦO2*1 , and ΦC*3 depend on both the sample’s BrC content and the light source’s irradiance.^14,21,23^ Third, while we developed predictive MLR models based on correlations between measurable BrC physicochemical properties and [^1^O_2_*]_ss_, [^3^C*]_ss_, ΦO2*1 , and ΦC*3 , interpreting the optical and photochemical behaviors of BrC ultimately requires connecting these properties and their propensity to produce ^1^O_2_* and ^3^C* to their molecular structures. Future work should consider combining spectroscopic measurements with other analytical methodologies capable of elucidating the molecular composition of BrC chromophores (e.g., high-resolution mass spectrometry). Fourth, while our OPLS-MLR approach will effectively model [^1^O_2_*]_ss_, [^3^C*]_ss_, ΦO2*1 , and ΦC*3 within a given study, its utility for creating universal models from aggregated, multistudy data sets is limited by interlaboratory variations in experimental conditions. Differences in methodology, such as light source spectra, will fundamentally affect the measured values of [^1^O_2_*]_ss_, [^3^C*]_ss_, ΦO2*1 , and ΦC*3 ,^14,21,23^ thereby obscuring their intrinsic relationships with BrC physicochemical properties. For instance, this study used narrow-band UVA irradiation, whereas quantum yields are wavelength-dependent.^21,64^ These spectral differences lead to deviations between studies and from true ambient solar conditions, preventing direct comparison across data sets. Machine learning (ML) models, with their capacity to handle complex, nonlinear interactions and process large, varied data sets, are well-suited to overcome this specific limitation.^57,65^ By learning from aggregated legacy data, ML algorithms can account for and generalize across the methodological “noise” introduced by interlaboratory differences, potentially revealing the robust, underlying relationships between BrC properties and oxidant production. Thus, future work should focus on applying ML to aggregated legacy data sets to account for nonlinearities and validate predictions of ^1^O_2_* and ^3^C* production by BrC across diverse experimental conditions.

## Supplementary Material



## Data Availability

The data and source codes can be accessed upon request (theodora.nah@cityu.edu.hk).

## Notes

Details of the method used to correct the inhibitory effects of WSOC on SYR oxidation by ^3^C* (Text S1); figures showing spectral irradiance in the photoreactor used for photochemical experiments and comparison with solar irradiance (Figure S1), violin plots showing the seasonal variations of (a) [^1^O_2_*]_ss_, (b) [^3^C*]_ss_, (c) ΦO2*1 , and (d) ΦC*3 for the aqueous extracts of PM_2.5_ samples (Figure S2), relative abundances of the PARAFAC-resolved components in the 28 aqueous extracts of PM_2.5_ samples arranged in order of a time series (Figure S3), absolute difference between actual and reconstructed product matrix *V* from NMF analysis (Figure S4), and results of OPLS analysis (Figure S5); tables showing the details of the aqueous extracts of PM_2.5_ samples used in this study (Table S1), absorbance and fluorescence parameters measured (Table S2), summary of [^1^O_2_*]_ss_, [^3^C*]_ss_, ΦO2*1 , and ΦC*3 used in this study (Table S3), and list of Spearman’s correlations between absorbance and fluorescence parameters and WSOC concentration and [^1^O_2_*]_ss_, [^3^C*]_ss_, ΦO2*1 , and ΦC*3 (Table S4) (PDF)
