# Supplementary material for: Connecting Brown Carbon Composition and Physicochemical Properties of Aqueous Urban PM2.5 to their Photosensitized Production of Singlet Oxygen and Organic Triplet Excited States
Source: Environ Sci Technol. 2026 Feb 14;60(8):6442–53. doi: 10.1021/acs.est.5c15686 (PMC12961739; doi:10.1021/acs.est.5c15686)
Supplement: Supplementary file 1 [file es5c15686_si_001.pdf]

Supplemental information for

Connecting Brown Carbon Composition and Physicochemical Properties of Aqueous Urban $PM_{2.5}$ to their Photosensitized Production of Singlet Oxygen and Organic Triplet Excited States

Yuting Lyu[a], Yitao Li[a,b], Ruihan Ma[a], Tianye Zhou[a], Nadine Borduas-Dedekind[c], Theodora Nah[a,b*]

[a]*School of Energy and Environment, City University of Hong Kong, Hong Kong SAR, China*

[b]*State Key Laboratory of Marine Environmental Health, City University of Hong Kong, Hong Kong SAR, China*

[c]*Department of Chemistry, University of British Columbia, Vancouver, BC V6T 1Z1, Canada*

** Correspondence: Theodora Nah (theodora.nah@cityu.edu.hk, Tel: +852 3442 5578, Postal address: School of Energy and Environment, Yeung Kin Man Academic Building, City University of Hong Kong, Tat Chee Avenue, Kowloon, Hong Kong)*

14 Pages, 1 Text, 5 Figures, 4 Tables

Text S1. Correction to the inhibitory effects of WSOC on SYR oxidation by $^3C^*$

In our previous study (Lyu et al., ACP, 2023), we detailed the quantification of $[^3C^*]_{ss}$, and $\Phi_{^3C^*}$ in $PM_{2.5}$ extracts.[1] Subsequent papers showed that WSOC in ambient BrC extracts can inhibit the oxidation of our probe, syringol (SYR), by $^3C^*$.[2, 3] To correct for this inhibitory effect, we used the approach detailed by Ma et al. (2024) and applied an empirical equation to calculate the inhibition factor (IF):[4]

$$IF_{SYR,corr} = 0.015 \times [WSOC] + 1 \quad \text{Eq. (1)}$$

where $IF_{SYR,corr}$ was the inhibition factor using SYR as the oxidizing $^3C^*$ probe, [WSOC] is the concentration of WSOC (mg-C L^{-1}). This factor was then applied to adjust the previously measured steady-state concentrations and quantum yields of $^3C^*$ by equation (2) and (3), respectively.

$$[^3C^*]_{ss,corr} = [^3C^*]_{ss,pre} / IF_{SYR,corr} \quad \text{Eq. (2)}$$

$$\Phi_{^3C^*,corr} = \Phi_{^3C^*,prev} / IF_{SYR,corr} \quad \text{Eq. (3)}$$

where $[^3C^*]_{ss,corr}$ and $\Phi_{^3C^*,corr}$ were the corrected steady-state concentrations and quantum yields of $^3C^*$, respectively, and $[^3C^*]_{ss,pre}$ and $\Phi_{^3C^*,prev}$ were the previously reported values, respectively.[1] Only the corrected values were used in the main text for various statistical analyses.

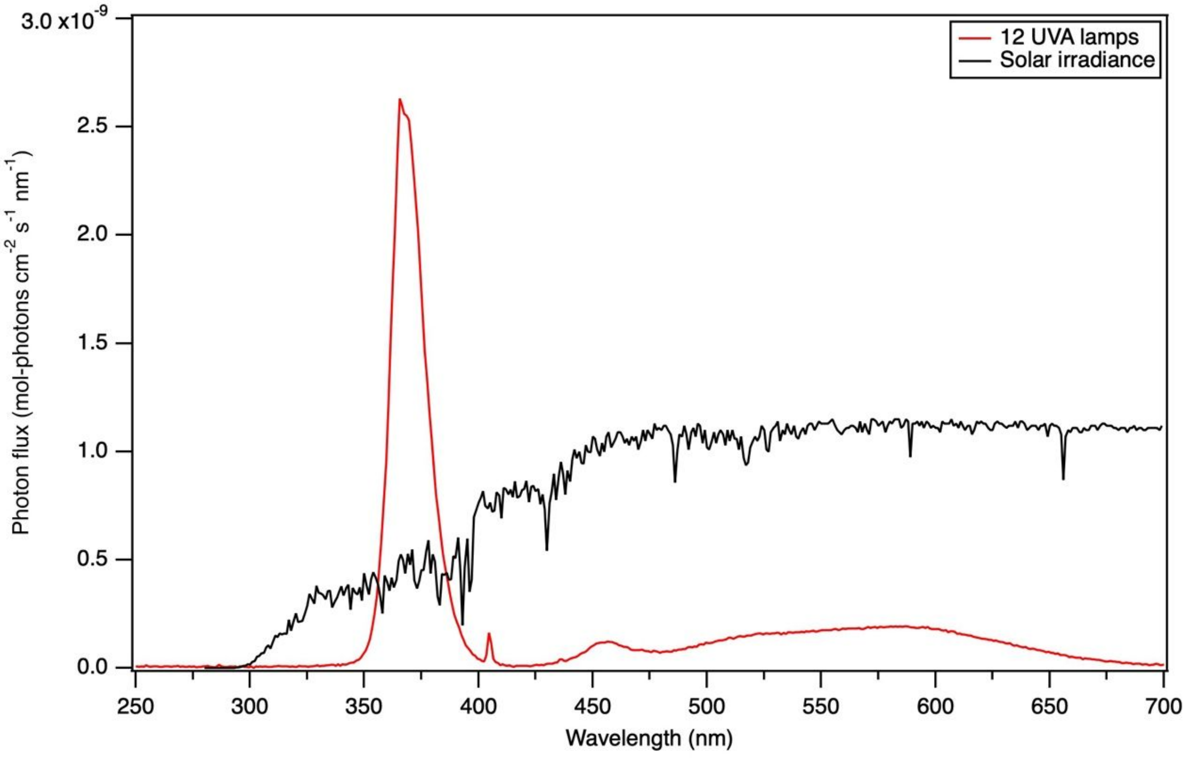

Figure S1. Spectral irradiance in the photoreactor (Rayonet RPR-3500A; Southern New England Ultraviolet Co.), and comparison with solar irradiance at Hong Kong on summer solstice at noon on 12/06/2021 (source: https://www.acom.ucar.edu/Models/TUV/Interactive_TUV/).

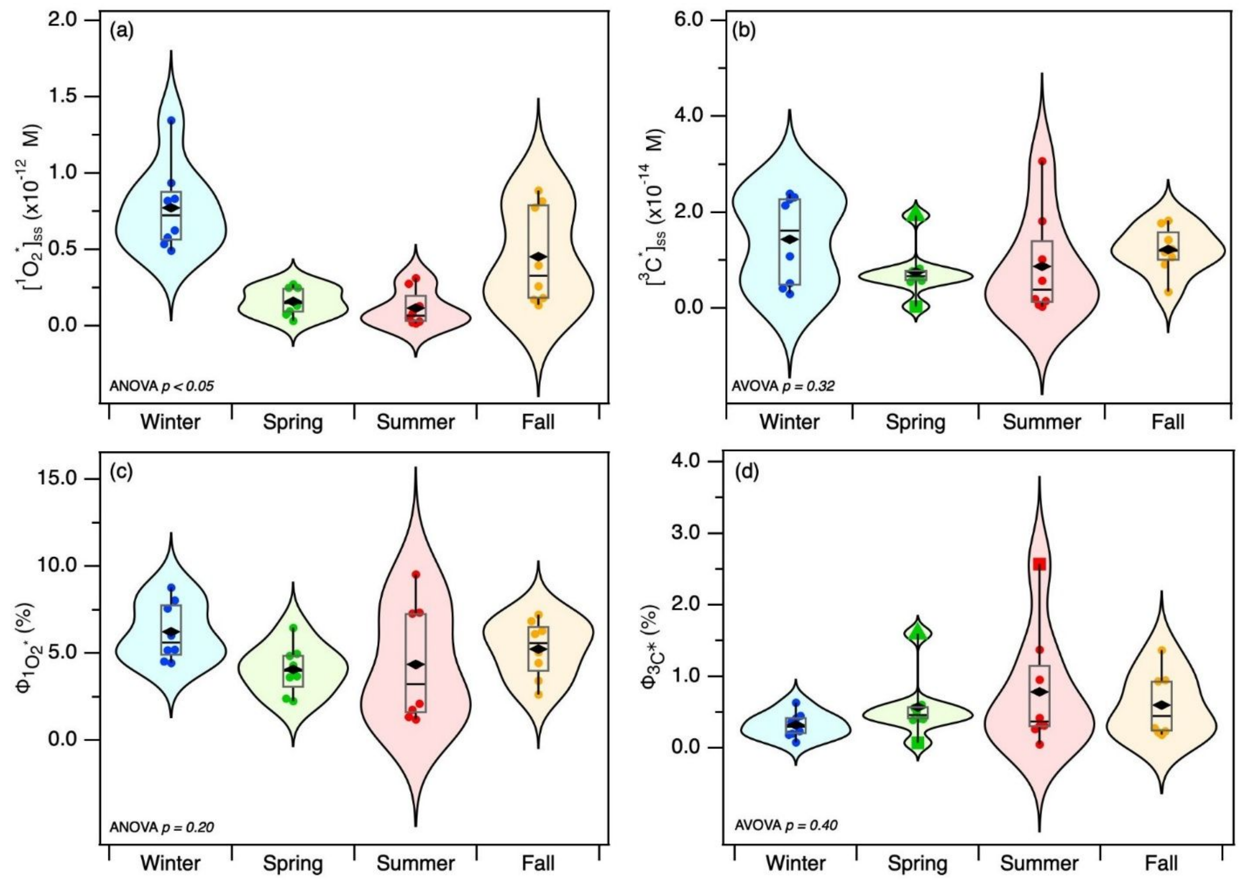

Figure S2. Violin plots showing the seasonal variations of (a) $[^1O_2^*]_{ss}$, (b) $[^3C^*]_{ss}$, (c) $\Phi_{^1O_2^*}$, and (d) $\Phi_{^3C^*}$ for the aqueous extracts of $PM_{2.5}$ samples. For the box plots, the triangles indicate far-out outliers, and the squares indicate outliers identified by Tukey's fences. The whiskers denote the minimum and maximum values, the boxes denote the 25th and 75th percentile values, black diamonds indicate the mean values, and the midlines of the boxes denote the median values.

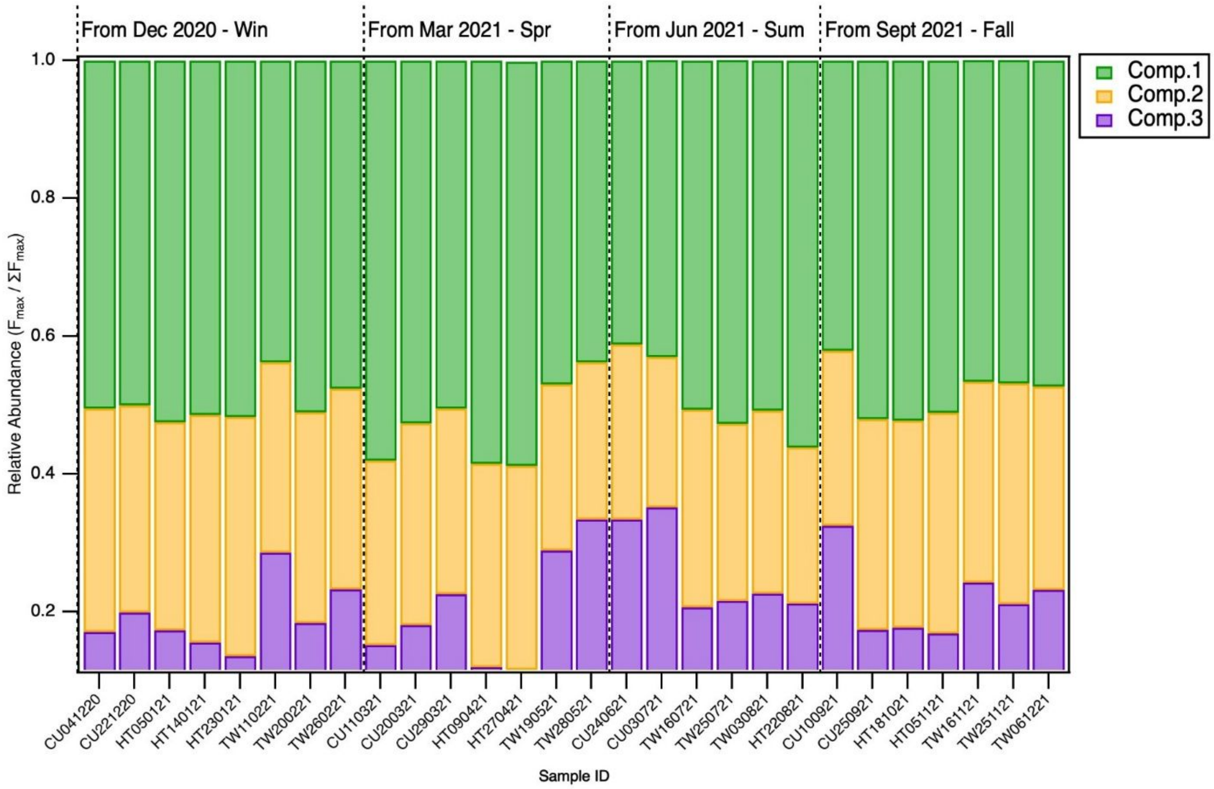

Figure S3. Relative abundances of the PARAFAC-resolved components in the 28 aqueous extracts of $PM_{2.5}$ samples arranged in order of a time series. See Table S1 for details on the $PM_{2.5}$ samples used in this study, including how the samples are named.

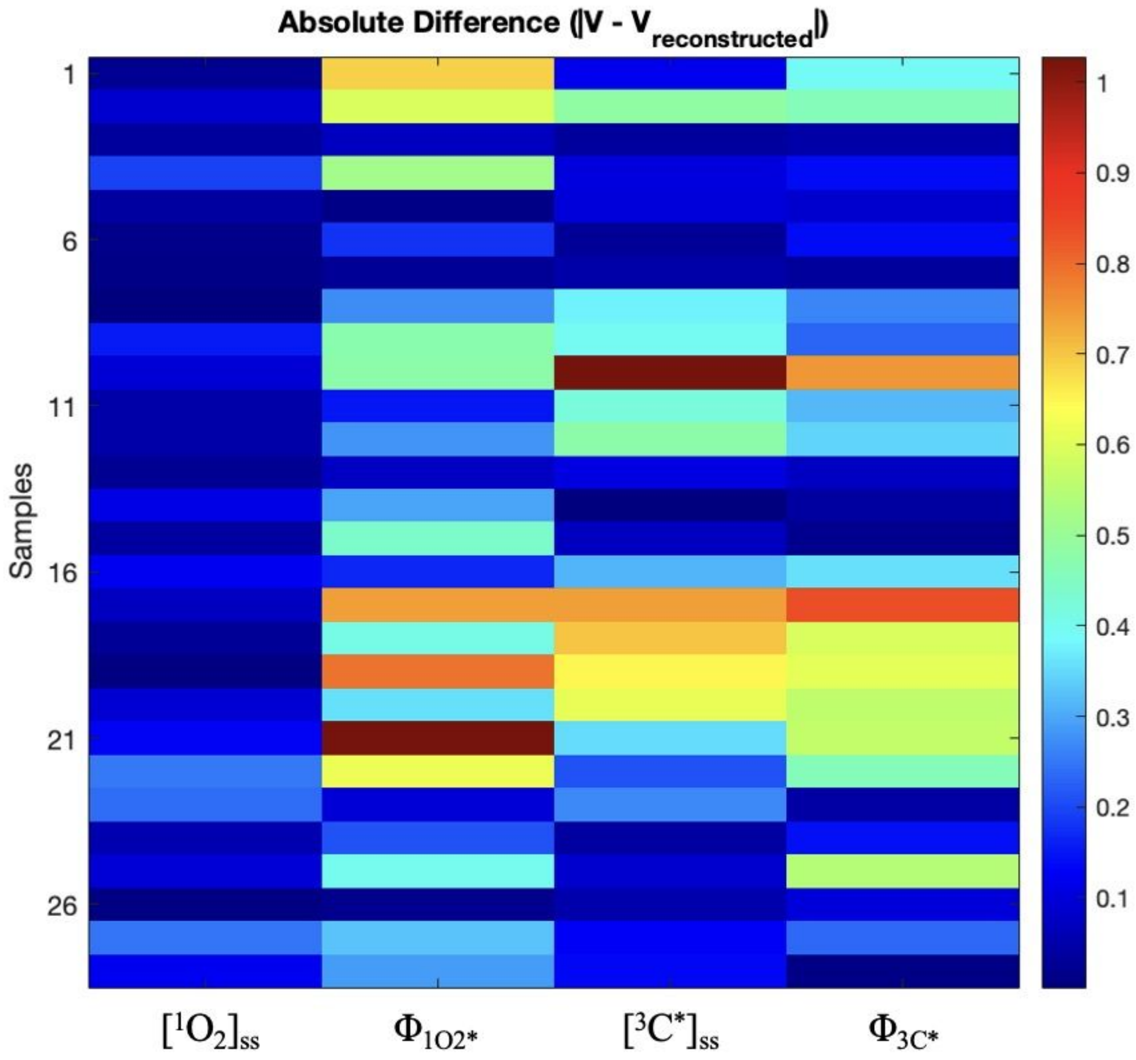

Figure S4. The absolute difference (i.e., residual matrix *D* in Eq. 1 in main text) between actual and re-constructed product matrix V from NMF analysis in MATLAB. The results indicated small errors in the calculation of three fluorescent components contributions to $[^1O_2^*]_{ss}$, $[^3C^*]_{ss}$, $\Phi_{^1O_2^*}$, and $\Phi_{^3C^*}$ (Figure 2). The left axis shows the numbers of 28 aqueous extract of $PM_{2.5}$ samples. Note that, to ensure good performance of NMF, the dataset was scaled to the range of 0 to 1 without changing the distribution of dataset.

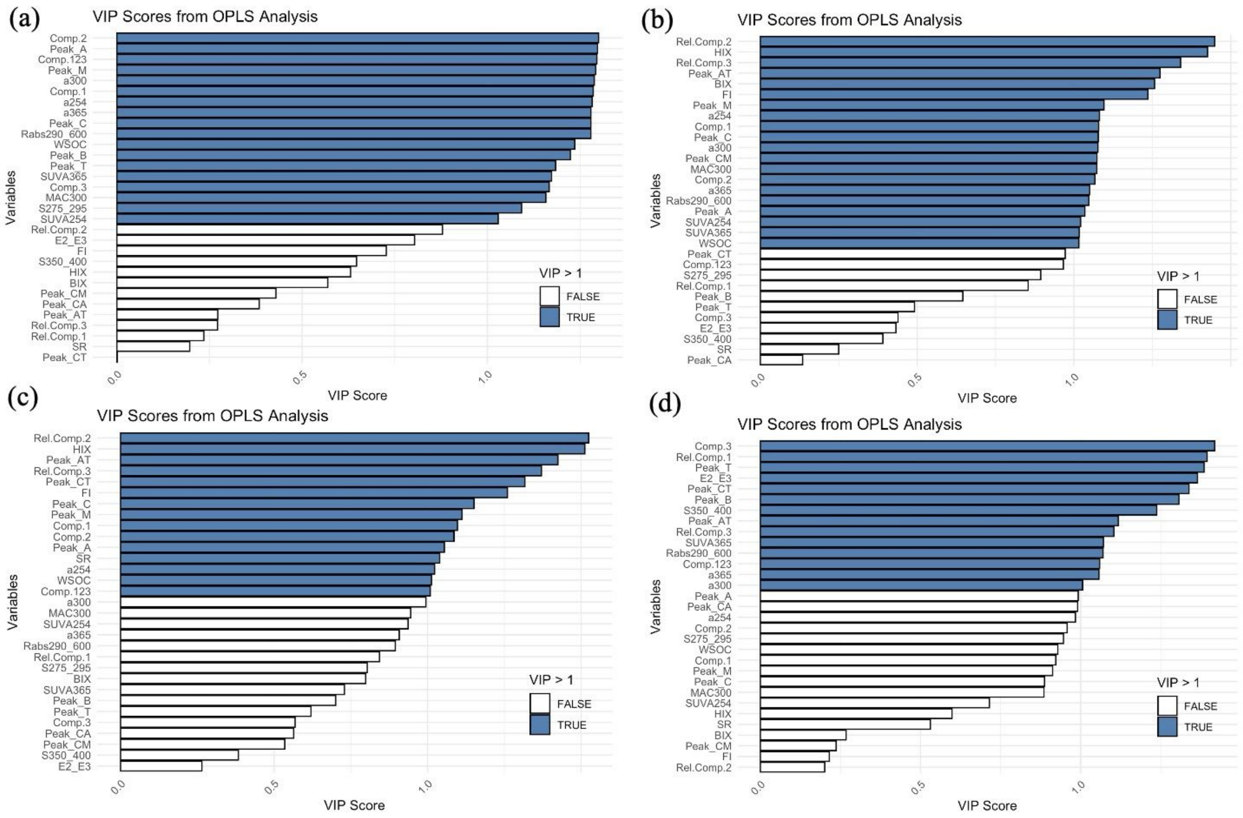

Figure S5. Results of OPLS on the parameters that were analyzed for (a) $[^1O_2^*]_{ss}$, (b) $[^3C^*]_{ss}$, (c) $\Phi_{^1O_2^*}$, and (d) $\Phi_{^3C^*}$. Only those with variable importance in the projection (VIP) score greater than 1.0 (shaded in blue) were selected for subsequent MLR analysis.

Table S1. Details of the aqueous extracts of $PM_{2.5}$ samples used in this study.

Season	CityU			Tsuen Wan			Hok Tsui		
	Sample ID[a]	Total sets[b]	Mass ratio[c]	Sample ID[a]	Total sets[b]	Mass ratio[c]	Sample ID[a]	Total sets[b]	Mass ratio[c]
Winter	CU041220	3	2.11	TW110221	3	1.43	HT050121	3	1.67
				TW200221	2	1.46	HT140121	3	2.01
	CU221220	2	1.68	TW260221	2	1.16	HT230121	3	1.47
Spring	CU110321	3	1.33	TW190521	3	0.49	HT090421	3	0.95
	CU200321	3	1.58	TW280521	3	0.71	HT270421	2	0.84
	CU290321	3	1.01						
Summer	CU240621	3	0.82	TW160721	3	0.86			
	CU030721	3	0.58	TW250721	3	1.08	HT220821	3	0.19
				TW030821	3	1.12			
Fall	CU100921	2	0.87	TW161121	3	1.71	HT181021	3	0.62
				TW251121	3	1.24			
	CU250921	3	1.48	TW061221	3	2.14	HT051121	3	0.69

Note: The CityU (CU) and Tsuen Wan (TW) sites are located in urban areas with many residential and commercial (and industrial for TW) activities. The semi-rural coastal Hok Tsui (HT) site is located away from local emission sources (approximately 6 km away from the closest urban area), which was mostly used as a receptor site to monitor air pollution originating from sources outside of Hong Kong in past studies (Tanner and Law, 2002; Li et al., 2018). More detailed information about sampling campaign and sample treatment can be found in Lyu et al. 2023.

a. The samples refer to the aqueous extracts of $PM_{2.5}$ that were collected on 47 mm quartz filters (Pall Tissuquartz™, 2500 QAT-UP). The IDs were defined as the sampling site followed by the sampling start date, e.g., CU041220 refers to $PM_{2.5}$ collected at the CityU site starting from 4 December 2020.

b. The aqueous extracts of $PM_{2.5}$ from two or three consecutive sampling sets (all filters in 6 days or 9 days, respectively) were aggregated to minimize daily variability and to better represent the seasonality. Each sample set was collected continuously for 3 days. For sample IDs that were comprised of three sets of filters (e.g., CU041220), this meant that the aggregated extracts were comprised of three consecutive 3-day sampling periods (9 days in total).

c. The $PM_{2.5}$ mass/water mass ratio (μg $PM_{2.5}$ /μg H_2O) was calculated by taking the ratio of the $PM_{2.5}$ mass divided by the water mass for each aggregated extract sample. The $PM_{2.5}$ mass was calculated using the daily $PM_{2.5}$ mass concentration measured at or near the sampling sites by Hong Kong Environmental Protection Department (HKEPD) (https://cd.epic.epd.gov.hk/EPICDI/air/station/?lang=en). Since the CityU site did not have a $PM_{2.5}$ mass monitor, the $PM_{2.5}$ mass concentration data at the closest HKEPD monitor site (Sham Shui Po, 1.5 km from CityU) was used to calculate the mass ratio for CityU samples. The $PM_{2.5}$ mass concentration data for Hok Tsui was not publicly available and had to be requested from the HKEPD. Since a consistent extraction protocol and constant dilution ratio were applied to each aggregated sample, the $PM_{2.5}$ mass to water mass ratios were calculated on a per filter basis. To obtain the $PM_{2.5}$ mass collected onto each filter, the 9-day or 6-day averaged $PM_{2.5}$ mass concentration was multiplied by the filter sampler's flow rate (we used 29 L min^{-1} in our calculations since the sampling flow rate decreased from of 30 L min^{-1} to 28 L min^{-1} over the 3-d continuous sampling period) and sampling time (72-h × 60 min). The mass ratios were calculated under the same conditions as in photochemical experiments (i.e., measurement of $^1O_2^*$ and $^3C^*$), which was equivalent to extracting each filter in 15.54 mL Milli-Q water. These values served as an upper bound due to materials lost during water extraction and filtration process.

Table S2. Absorbance and fluorescence parameters measured. Also shown are their definitions, calculation methods, and interpretations as compiled from literature.[5-11]

**Parameter**	**Calculation**	**Description**
α_{254}, α_{300}, α_{365}	Directly read from the absorption spectra at wavelengths of 254 nm, 300 nm, and 365 nm, respectively.	UV absorption coefficients at 254 nm, 300 nm, and 365 nm, respectively.
R_{abs}	$R_{abs} = \frac{10^3}{d}\sum_{290\,nm}^{600\,nm} I_{0,\lambda}(1-10^{-a_\lambda d})\Delta\lambda$	The R_{abs} denotes the total light absorbance rate of a sample in the range of 290 and 600 nm.
MAC_{300}	$\mathrm{MAC}_{300} = \frac{\text{Absorbance at 300 nm}}{[\mathrm{WSOC}] \times l}$ MAC_{300} is the WSOC-normalized absorbance coefficient. [WSOC] is the extracted water-soluble organic concentration. l is the pathlength (1 cm).	MAC_{300} can be used as an indication of the degree of aromaticity for the chromophores. A larger MAC_{300} value indicates a higher degree of aromaticity.
$SUVA_{254}$	$\mathrm{SUVA}_{254} = \frac{\text{Absorbance at 254 nm}}{[\mathrm{WSOC}] \times l}$ $SUVA_{254}$ is the WSOC-normalized absorbance coefficient. [WSOC] is the extracted water-soluble organic concentration. l is the pathlength (1 cm).	$SUVA_{254}$ can be used as an indication of the degree of aromaticity for the chromophores. A larger $SUVA_{254}$ value indicates a higher degree of aromaticity.
$SUVA_{365}$	$\mathrm{SUVA}_{365} = \frac{\text{Absorbance at 365 nm}}{[\mathrm{WSOC}] \times l}$ $SUVA_{254}$ is the WSOC-normalized absorbance coefficient. [WSOC] is the extracted water-soluble organic concentration. l is the pathlength (1 cm).	SUVA365 can be used as an indication of the degree of aromaticity for the chromophores. A larger $SUVA_{365}$ value indicates a higher degree of aromaticity.
E_2/E_3	$\mathrm{E}_2/\mathrm{E}_3 = \frac{\text{Absorbance at 250 nm}}{\text{Absorbance at 365 nm}}$ E_2/E_3 is the ratio of the absorbance at 250 nm to the absorbance at 365 nm.	The E_2/E_3 ratio denotes the molecular weights of chromophores. A larger E_2/E_3 value indicates smaller molecular weight.
$S_{275\text{-}295}$	$S_{275-295} = a(\lambda_{275})e^{-S(\lambda_{295}-\lambda_{275})}$, $S_{275\text{-}295}$ is the spectral slope from 275 nm to 295 nm.	$S_{275\text{-}295}$ denotes the aromaticity and/or molecular weight of organic species. Larger values indicate lower aromaticity and/or smaller molecular weight.
$S_{350\text{-}400}$	$S_{350-400} = a(\lambda_{350})e^{-S(\lambda_{400}-\lambda_{350})}$ $S_{350\text{-}400}$ is the spectral slope from 350 nm to 400 nm.	$S_{350\text{-}400}$ denotes the aromaticity and/or molecular weight of organic species. Larger values indicate lower aromaticity and/or smaller molecular weight.

S_R	$$S_R = \frac{S_{275-295}}{S_{350-400}}$$ S_R is the ratio of $S_{275\text{-}295}$ to $S_{350\text{-}400}$.	S_R negatively correlates with the molecular weight of organic species.
FI	$$\text{FI} = \frac{I(E_x = 370\text{ nm}, E_m = 450\text{ nm})}{I(E_x = 370\text{ nm}, E_m = 500\text{ nm})}$$ FI is the fluorescence index.	FI can be used to determine the aromaticity of fluorophores. A smaller FIX value indicates a higher degree of aromaticity.
BIX	$$\text{BIX} = \frac{\text{I}(E_x = 310\text{nm}, E_m = 380\text{ nm})}{\text{I}(E_x = 310\text{nm}, E_m = 430\text{ nm})}$$ BIX is the biological index.	BIX denotes the influence of biological activities in aquatic environments, but it is also negatively correlated with the aromaticity and molecular weight of organic matter.
HIX	$$\text{HIX} = \frac{\text{I}(E_x = 255\text{ nm},\ E_m = 435 - 480\text{nm})}{\text{I}(E_x = 355\text{ nm},\ E_m = 300 - 345\text{nm})}$$ HIX is the humidification index.	HIX denotes the extent of humidification. A larger HIX value indicates a higher degree of humification or a larger abundance of humic substances.
Peak A	λ_{ex} = 240 – 295 nm, λ_{em} = 390 – 500 nm	Peak A denotes humic-like substances (HULIS).
Peak C	λ_{ex} = 290 – 355 nm, λ_{em} = 380 – 480 nm	Peak C denotes fulvic acid.
Peak M	λ_{ex} = 290 – 315 nm, λ_{em} = 370 – 420 nm	Peak M denotes marine-derived humic-like substances.
Peak B	λ_{ex} = 270 – 280 nm, λ_{em} = 300 – 320 nm	Peak B denotes tyrosine-like organic matter.
Peak T	λ_{ex} = 240 – 275 nm, λ_{em} = 325 – 330 nm	Peak T denotes tryptophan-like organic matter.
Peak A:T	The ratio of Peak A intensity to Peak T intensity	Relative abundance of HULIS to tryptophan-like fluorescent compounds.
Peak C:T	The ratio of Peak C intensity to Peak T intensity	Relative abundance of fulvic acid to tryptophan-like fluorescent compounds.
Peak C:A	The ratio of Peak C intensity to Peak A intensity	Relative abundance of fulvic acid to HULIS.
Peak C:M	The ratio of Peak C intensity to Peak M intensity	Relative abundance of fulvic acid to marine-derived HULIS.
Comp. 1	Maximum fluorescence intensity of the identified fluorescent component 1 (F_{max1}).	The absolute intensity of less oxygenated compounds from BB

		as identified by PARAFAC model analysis.
Comp. 2	Maximum fluorescence intensity of the identified fluorescent component 2 (F_{max2}).	The absolute intensity of highly oxygenated compounds as identified by PARAFAC model analysis.
Comp. 3	Maximum fluorescence intensity of the identified fluorescent component 3 (F_{max3}).	The absolute intensity of aromatic derivatives as identified by PARAFAC model analysis.
Comp. 123	$\sum F_{max} = F_{max1} + F_{max2} + F_{max3}$	The sum of absolute intensities of fluorescent components 1, 2, and 3.
Rel. Comp. 1	Rel. Comp. 1 = $F_{max1} / \sum F_{max}$	The relative intensity of fluorescent component 1.
Rel. Comp. 2	Rel. Comp. 2 = $F_{max2} / \sum F_{max}$	The relative intensity of fluorescent component 2.
Rel. Comp. 3	Rel. Comp. 3 = $F_{max3} / \sum F_{max}$	The relative intensity of fluorescent component 3.

Note: For simplicity, we refer to Comp. 1, Comp. 2, and Comp. 3 as ASOA+BSOA, HO-OA, and LMW-AA components, respectively, in the main text. Additionally, we refer to Rel. Comp. 1, Rel. Comp. 2, and Rel. Comp. 3 as Rel. ASOA+BSOA, Rel. HO-OA, and Rel. LMW-AA components, respectively, in the main text. Comp. 123 is referred to as FluoresComp. sum in the main text.

Table S3. Summary of $[^1O_2^*]_{ss}$, $[^3C^*]_{ss}$, $\Phi_{^1O_2^*}$, and $\Phi_{^3C^*}$ for the aqueous extracts of $PM_{2.5}$ samples used in this study. Seasonal variations of $[^1O_2^*]_{ss}$, $[^3C^*]_{ss}$, $\Phi_{^1O_2^*}$, and $\Phi_{^3C^*}$ are shown in Figure S1.

Sample ID	$[^1O_2^*]_{ss}$ ($\times 10^{-13}$ M)	$\Phi_{^1O_2^*}$ (%)	$[^3C^*]_{ss}$ ($\times 10^{-15}$ M)	$\Phi_{^3C^*}$ (%)
CU041220	8.21 ± 1.02	5.20 ± 0.62	22.72 ± 9.76	0.45 ± 0.20
CU221220	6.27 ± 0.89	4.45 ± 0.56	10.89 ± 4.83	0.23 ± 0.11
CU110321	1.64 ± 0.25	3.68 ± 0.46	6.51 ± 3.23	0.41 ± 0.21
CU200321	2.51 ± 0.46	2.24 ± 0.30	19.33 ± 8.97	0.51 ± 0.25
CU290321	2.49 ± 0.38	4.31 ± 0.53	8.32 ± 3.94	0.42 ± 0.21
CU240621	1.28 ± 0.33	4.34 ± 0.76	10.27 ± 5.20	0.95 ± 0.50
CU030721	0.16 ± 0.06	1.19 ± 0.31	1.54 ± 0.80	0.32 ± 0.17
CU100921	2.59 ± 0.34	6.29 ± 0.78	3.48 ± 1.69	0.24 ± 0.12
CU250921	3.98 ± 0.55	7.21 ± 0.88	17.81 ± 8.42	0.93 ± 0.46
TW110221	5.80 ± 0.65	5.18 ± 0.57	3.06 ± 1.37	0.08 ± 0.04
TW200221	4.92 ± 0.65	6.03 ± 0.69	5.22 ± 2.46	0.19 ± 0.09
TW260221	5.37 ± 0.65	8.78 ± 1.03	4.23 ± 1.94	0.20 ± 0.10
TW190521	0.33 ± 0.08	2.41 ± 0.40	0.38 ± 0.20	0.07 ± 0.04
TW280521	2.73 ± 0.33	4.85 ± 0.56	7.77 ± 3.73	0.40 ± 0.20
TW160721	2.78 ± 0.28	7.27 ± 0.80	5.82 ± 2.87	0.43 ± 0.22
TW250721	0.80 ± 0.32	2.11 ± 0.36	18.15 ± 8.67	1.37 ± 0.69
TW030821	3.14 ± 0.43	9.54 ± 1.18	30.73 ± 15.48	2.57 ± 1.35
TW161121	7.76 ± 0.86	6.11 ± 0.67	9.13 ± 3.93	0.22 ± 0.10
TW251121	8.17 ± 0.95	4.44 ± 0.49	10.69 ± 4.38	0.19 ± 0.08
TW061221	8.88 ± 1.07	6.83 ± 0.77	11.68 ± 4.93	0.28 ± 0.11
HT050121	9.37 ± 1.27	4.53 ± 0.54	23.87 ± 10.16	0.36 ± 0.16
HT140121	13.47 ± 1.50	7.59 ± 0.87	23.14 ± 9.72	0.41 ± 0.18
HT230121	8.33 ± 1.34	8.03 ± 1.19	21.50 ± 9.42	0.64 ± 0.29
HT090421	1.34 ± 0.28	4.97 ± 0.83	5.74 ± 2.78	0.61 ± 0.31
HT270421	0.76 ± 0.30	6.47 ± 1.51	6.89 ± 3.52	1.60 ± 0.85
HT220821	0.23 ± 0.07	1.35 ± 0.33	1.98 ± 1.03	0.32 ± 0.17
HT181021	1.38 ± 0.42	3.43 ± 0.49	18.31 ± 8.23	1.37 ± 0.64

HT051121	1.72 ± 0.58	2.62 ± 0.59	14.27 ± 6.99	0.61 ± 0.31

Note: $[^3C^*]_{ss}$ and $\Phi_{^3C^*}$ were corrected from the previously reported values in Lyu et al. (2023)[1] as described in Text S1.

Table S4. List of Spearman's correlations between absorbance and fluorescence parameters and WSOC concentration and $[^1O_2^*]_{ss}$, $[^3C^*]_{ss}$, $\Phi_{^1O_2^*}$, and $\Phi_{^3C^*}$ for the aqueous extracts of $PM_{2.5}$ samples. Yellow highlighted parameters are those found to have statistical significance.

Ranking	$[^1O_2^*]_{ss}$	*r*	$[^3C^*]_{ss}$	*r*	$\Phi_{^1O_2^*}$	*r*	$\Phi_{^3C^*}$	*r*
1	α_{300}	*0.91*	Rel.Comp. 2	*0.59*	Rel.Comp. 2	*0.39*	Rel.Comp. 1	*0.54*
2	α_{254}	*0.90*	Peak A	*0.54*	Comp. 2	*0.38*	Peak C:T	*0.50*
3	α_{365}	*0.90*	WSOC	*0.53*	Peak C	*0.35*	Peak C:A	*0.47*
4	R_{abs}	*0.89*	Peak M	*0.53*	HIX	*0.35*	Peak A:T	*0.43*
5	Comp. 2	*0.88*	Comp. 2	*0.52*	Peak A	*0.34*	HIX	*0.26*
6	WSOC	*0.87*	Comp. 1	*0.52*	WSOC	*0.34*	Rel.Comp. 2	*0.18*
7	Peak A	*0.86*	HIX	*0.51*	α_{254}	*0.33*	Peak C:M	*0.17*
8	Comp. 123	*0.86*	Comp. 123	*0.50*	Comp. 123	*0.33*	E_2/E_3	*0.16*
9	Peak M	*0.85*	α_{365}	*0.50*	α_{300}	*0.32*	$S_{350\text{-}400}$	*0.14*
10	Comp. 1	*0.85*	R_{abs}	*0.49*	Comp. 1	*0.31*	$S_{275\text{-}295}$	*0.10*
11	Peak C	*0.84*	Peak C	*0.49*	Peak M	*0.30*	S_R	*-0.09*
12	$SUVA_{365}$	*0.81*	Peak A:T	*0.49*	Peak B	*0.29*	WSOC	*-0.16*
13	MAC_{300}	*0.81*	MAC_{300}	*0.48*	Peak T	*0.28*	Peak C	*-0.19*
14	Peak B	*0.81*	$SUVA_{365}$	*0.48*	MAC_{300}	*0.27*	BIX	*-0.19*
15	Peak T	*0.80*	α_{300}	*0.47*	α_{365}	*0.27*	Peak A	*-0.19*
16	Comp. 3	*0.78*	α_{254}	*0.46*	Comp. 3	*0.27*	Comp. 2	*-0.20*
17	$SUVA_{254}$	*0.72*	Peak C:M	*0.44*	R_{abs}	*0.25*	Peak M	*-0.21*
18	Rel.Comp. 2	*0.65*	Peak C:T	*0.43*	$SUVA_{254}$	*0.25*	Comp.1	*-0.21*
19	HIX	*0.45*	$SUVA_{254}$	*0.39*	Peak C:M	*0.21*	MAC_{300}	*-0.22*
20	Peak C:M	*0.33*	Peak B	*0.35*	$SUVA_{365}$	*0.19*	Comp. 123	*-0.24*
21	Peak A:T	*0.23*	Rel.Comp. 1	*0.30*	Peak C:T	*0.16*	$SUVA_{254}$	*-0.24*
22	Peak C:T	*0.15*	Peak T	*0.30*	Peak A:T	*0.15*	$SUVA_{365}$	*-0.27*
23	Rel.Comp. 1	*-0.17*	Comp. 3	*0.30*	Peak C:A	*0.03*	α_{254}	*-0.28*
24	Rel.Comp. 3	*-0.18*	Peak C:A	*-0.04*	E_2/E_3	*0.01*	α_{300}	*-0.28*
25	S_R	*-0.24*	S_R	*-0.22*	$S_{350\text{-}400}$	*-0.07*	α_{365}	*-0.29*
26	Peak C:A	*-0.25*	Rel.Comp. 3	*-0.44*	Rel.Comp. 1	*-0.08*	R_{abs}	*-0.29*
27	BIX	*-0.47*	$S_{350\text{-}400}$	*-0.47*	Rel.Comp. 3	*-0.16*	FI	*-0.30*
28	FI	*-0.50*	E_2/E_3	*-0.51*	BIX	*-0.20*	Peak B	*-0.36*
29	$S_{350\text{-}400}$	*-0.67*	FI	*-0.58*	FI	*-0.26*	Peak T	*-0.40*
30	E_2/E_3	*-0.67*	BIX	*-0.59*	$S_{275\text{-}295}$	*-0.26*	Comp. 3	*-0.41*
31	$S_{275\text{-}295}$	*-0.81*	$S_{275\text{-}295}$	*-0.61*	S_R	*-0.30*	Rel.Comp. 3	*-0.47*

Note: For simplicity, we refer to Comp. 1, Comp. 2, and Comp. 3 as ASOA+BSOA, HO-OA, and LMW-AA components, respectively, in the main text. Additionally, we refer to Rel. Comp. 1, Rel. Comp. 2, and Rel. Comp. 3 as Rel. ASOA+BSOA, Rel. HO-OA, and Rel. LMW-AA components, respectively, in the main text. Comp. 123 is referred to as FluoresComp. sum in the main text.

References

1. Lyu, Y.; Lam, Y. H.; Li, Y.; Borduas-Dedekind, N.; Nah, T., Seasonal variations in the production of singlet oxygen and organic triplet excited states in aqueous PM2.5 in Hong Kong SAR, South China. *Atmos. Chem. Phys.* **2023,** *23*, (16), 9245-9263.
2. Ma, L.; Worland, R.; Tran, T.; Anastasio, C., Evaluation of Probes to Measure Oxidizing Organic Triplet Excited States in Aerosol Liquid Water. *Environ Sci Technol* **2023,** *57*, (15), 6052-6062.
3. Ma, L.; Worland, R.; Jiang, W. Q.; Niedek, C.; Guzman, C.; Bein, K. J.; Zhang, Q.; Anastasio, C., Predicting photooxidant concentrations in aerosol liquid water based on laboratory extracts of ambient particles. *Atmos Chem Phys* **2023,** *23*, (15), 8805-8821.
4. Ma, L.; Worland, R.; Heinlein, L.; Guzman, C.; Jiang, W. Q.; Niedek, C.; Bein, K. J.; Zhang, Q.; Anastasio, C., Seasonal variations in photooxidant formation and light absorption in aqueous extracts of ambient particles. *Atmos Chem Phys* **2024,** *24*, (1), 1-21.
5. Cao, X. B.; Liu, J. M.; Wu, Y. L.; Cheng, Y.; Zheng, M.; He, K. B., A Review on Brown Carbon Aerosol in China: From Molecular Composition to Climate Impact. *Curr Pollut Rep* **2024,** *10*, (2), 326-343.
6. Jiang, H. X.; Li, J.; Sun, R.; Tian, C. G.; Tang, J.; Jiang, B.; Liao, Y. H.; Chen, C. E.; Zhang, G., Molecular Dynamics and Light Absorption Properties of Atmospheric Dissolved Organic Matter. *Environ Sci Technol* **2021,** *55*, (15), 10268-10279.
7. Korak, J. A.; McKay, G., Critical review of fluorescence and absorbance measurements as surrogates for the molecular weight and aromaticity of dissolved organic matter. *Environ Sci-Proc Imp* **2024,** *26*, (10), 1663-1702.
8. Wu, G. M.; Fu, P. Q.; Ram, K.; Song, J. Z.; Chen, Q. C.; Kawamura, K.; Wan, X.; Kang, S. C.; Wang, X. P.; Laskin, A.; Cong, Z. Y., Fluorescence characteristics of water-soluble organic carbon in atmospheric aerosol. *Environ Pollut* **2021,** *268*.
9. Lee, H. J.; Laskin, A.; Laskin, J.; Nizkorodov, S. A., Excitation-Emission Spectra and Fluorescence Quantum Yields for Fresh and Aged Biogenic Secondary Organic Aerosols. *Environ Sci Technol* **2013,** *47*, (11), 5763-5770.
10. Zhou, Y. Q.; Shi, K.; Zhang, Y. L.; Jeppesen, E.; Liu, X. H.; Zhou, Q. C.; Wu, H. W.; Tang, X. M.; Zhu, G. W., Fluorescence peak integration ratio IC:IT as a new potential indicator tracing the compositional changes in chromophoric dissolved organic matter. *Sci Total Environ* **2017,** *574*, 1588-1598.
11. Wasswa, J.; Driscoll, C. T.; Zeng, T., Photochemical Characterization of Surface Waters from Lakes in the Adirondack Region of New York. *Environ Sci Technol* **2020,** *54*, (17), 10654-10667.
